# Estimation of Contact PPG-Derived Vascular Indices from Reconstructed rPPG Signals

**DOI:** 10.3390/s26206458

**Published:** 2026-10-12

**Authors:** SiJin Kim, Seungkeon Lee, Saelim Jeong, Hakjin Lee, Kyong Keun Choi, Eui Chul Lee

**Affiliations:** 1 Department of Sports ICT Convergence, Graduate School, Sangmyung University, Hongjimun 2-Gil 20, Jongno-Gu, Seoul 03016, Republic of Korea; 202531030@sangmyung.kr; 2 Department of AI & Informatics, Graduate School, Sangmyung University, Hongjimun 2-Gil 20, Jongno-Gu, Seoul 03016, Republic of Korea; 202233053@sangmyung.kr (S.L.); 202431056@sangmyung.kr (S.J.); 202531048@sangmyung.kr (H.L.); 3 The Graduate School of Business Administration, Sangmyung University, Hongjimun 2-Gil 20, Jongno-Gu, Seoul 03016, Republic of Korea; 4 Department of Human-Centered AI, Sangmyung University, Hongjimun 2-Gil 20, Jongno-Gu, Seoul 03016, Republic of Korea

**Keywords:** signal processing, deep learning, pulse-wave analysis, vascular biomarkers, remote photoplethysmography

## Abstract

Remote photoplethysmography (rPPG) enables contactless cardiovascular monitoring using conventional cameras; however, its smooth waveform morphology limits the extraction of vascular indices that depend on fine pulse-wave characteristics. Consequently, most existing rPPG studies have focused primarily on heart rate estimation rather than morphological vascular assessment. This study investigates whether reconstructed signals can support the computation of contact-PPG (cPPG)-derived vascular indices and examines how agreement varies across timing-, amplitude-, and derivative-based indices. Fiducial and second derivative photoplethysmography (SDPPG) candidates were extracted from reconstructed signals to compute the Stiffness Index (SI), Reflection Index (RI), Augmentation Index (AIx), and an exploratory Simplified Aging Index (sAI) based on the detector-returned a, b, and e candidates. The resulting indices were compared with those derived from simultaneously acquired reference cPPG within pre-segmented, temporally paired cardiac cycles with known cycle boundaries. Reference cPPG fiducial locations were not used for representative window selection or reconstructed-signal fiducial localization. Reconstruction increased the number of cycles for which the adopted detector returned the candidates required for index computation. However, a detector audit showed that strict post-notch local maxima were present in only 5.02% of reconstructed cycles, indicating that candidate availability should not be interpreted as consistent recovery of a physiologically distinct diastolic peak. Under leave-one-subject-out cross-validation across all 28 participants, subject-level agreement under the adopted computational fiducial definition was highest for SI (r=0.963, ICC(2,1) =0.926) and for the height-independent, detector-defined systolic-to-post-notch delay (r=0.909, ICC(2,1) =0.837). An exploratory strict-local-peak sensitivity analysis retained only 37 matched cycles from four participants and descriptively showed weaker agreement than the fallback-inclusive analysis. Because this small subset contained repeated cycles from only four participants, it was considered insufficient for population-level inference and was not used to support subject-level claims regarding physiological diastolic-peak recovery. RI and AIx showed only weak associations whose 95% confidence intervals included zero, and sAI showed no meaningful agreement. Under the tested additive-noise conditions, detector-defined timing measures changed relatively little between the clean and 10 dB conditions, whereas amplitude-related measures and dicrotic notch localization degraded more markedly. Additional narrowing of the retained upper cutoff below 4 Hz produced relatively small changes, but wider-band conditions could not be evaluated using the stored signals. These findings support the use of cPPG-derived vascular indices as downstream measures for technically evaluating reconstructed rPPG morphology, but they do not constitute clinical validation or establish standalone non-contact vascular assessment.

## 1. Introduction

Photoplethysmography (PPG) is a representative biosignal that enables non-invasive assessment of cardiovascular status by measuring optical properties associated with blood volume changes [1]. Remote photoplethysmography (rPPG), which acquires cardiovascular signals from conventional camera videos, has recently attracted considerable attention [2,3]. While rPPG offers the advantage of camera-based, non-contact biosignal acquisition, its waveforms are generally smoother than those of contact photoplethysmography (cPPG), making it difficult to preserve fine morphological features such as the dicrotic notch and diastolic peak. Consequently, although rPPG has been widely applied to physiological measurements such as heart rate estimation, deriving SDPPG-based cPPG-derived vascular indices directly from raw rPPG signals remains challenging. To date, most rPPG studies have primarily focused on heart rate estimation or waveform reconstruction, and relatively few have investigated whether reconstructed signals preserve vascular morphology required for vascular index estimation. Moreover, existing reconstruction methods [4,5] have generally been evaluated using waveform similarity metrics, which do not necessarily indicate whether the morphological characteristics required for SDPPG-based vascular analysis are preserved. The objective of this study is therefore to investigate whether cPPG-derived vascular indices, traditionally computed from contact PPG, can also be estimated from reconstructed signals, recognizing that the degree of correspondence may vary across indices. SDPPG feature points [6] are extracted from the reconstructed signals to compute cPPG-derived vascular indices, including an exploratory Simplified Aging Index (sAI) computed from the detector-returned *a*, *b*, and *e* candidates instead of the conventional five-point (*a*–*e*) formulation [6,7], Stiffness Index (SI), Reflection Index (RI), and Augmentation Index (AIx) [8,9]. These indices are subsequently compared with those derived from simultaneously acquired reference cPPG to evaluate the extent to which reconstructed signals retain timing-, amplitude-, and derivative-domain characteristics under a defined downstream fiducial-extraction pipeline.

## 2. Related Works

### 2.1. rPPG-to-cPPG Signal Reconstruction

While rPPG offers the advantage of contactless biosignal acquisition, its pulsatile component tends to be weaker and its waveforms smoother compared to contact-based PPG, making it difficult to obtain fine morphological information. To address this limitation, various approaches have been proposed in prior work to enhance the signal quality of rPPG or to reconstruct it into a form closer to contact PPG. In particular, Kim et al. proposed a method that learns the correspondence between rPPG and cPPG to restore remote signals to the fidelity of contact-based signals [4], demonstrating the potential to leverage waveform morphology beyond simple heart rate estimation. Subsequent deep learning-based rPPG studies have explored reconstructing iPPG or rPPG signals acquired from cameras into more precise PPG waveforms, as well as utilizing the reconstructed signals for downstream biosignal analyses such as blood pressure estimation [5]. This line of research is significant in that it extends beyond heart rate-centric analysis toward waveform-level reconstruction. However, most existing studies have evaluated reconstructed signals primarily using waveform similarity metrics, such as the Pearson correlation coefficient (PCC), cosine similarity, root mean square error (RMSE), or heart rate estimation accuracy [4,10,11,12]. Recent work has directly investigated detailed rPPG waveform recovery, including the systolic peak, dicrotic notch, diastolic peak, and overall pulse-wave contour [13]. These studies demonstrate progress in morphology-aware waveform reconstruction; however, waveform-level similarity or fiducial visibility does not necessarily establish the validity of vascular indices derived from reconstructed waveforms. The contribution of the present study therefore does not lie in proposing a new reconstruction architecture but in evaluating an existing reconstruction model at the downstream physiological-index level by separately examining candidate availability, fiducial validity, numerical index error, robustness, subject-wise generalization, and cross-dataset transfer.

### 2.2. SDPPG-Based Morphological Analysis and Vascular Indices

Contact PPG waveforms provide vascular morphological information from both the original pulse waveform and its first and second derivatives [14]. The second derivative photoplethysmogram (SDPPG) is conventionally characterized by five fiducial points denoted as a, b, c, d, and e, and their relative amplitudes have been investigated in relation to vascular aging and arterial properties [6,7]. The clinical and physiological motivations for the indices evaluated in this study differ according to their measurement domains. SI is a height-dependent, timing-derived surrogate related to arterial stiffness; RI describes the relative amplitude of the secondary pulse-wave component; conventional AIx describes augmentation-related pulse-wave morphology; and SDPPG amplitude ratios have been investigated as markers associated with vascular aging. These measures should nevertheless be regarded as PPG-derived surrogates and should not be treated as interchangeable with direct clinical measurements such as carotid–femoral pulse-wave velocity or pressure-wave augmentation index [9].

When the secondary pulse-wave component or reflected-wave morphology is attenuated, the localization of post-systolic and SDPPG fiducial points may become unstable, thereby compromising the reliability of the derived indices. Downstream evaluation of reconstructed rPPG should therefore distinguish candidate availability, physiological landmark validity, and numerical agreement of the resulting indices rather than relying only on waveform similarity or apparent fiducial visibility.

## 3. Materials and Methods

### 3.1. Dataset

This study utilized a self-collected dataset comprising 30 participants aged 20 to 28 years (16 males and 14 females), from whom facial video and contact cPPG signals were simultaneously acquired. Before recording, participants completed a structured questionnaire covering age, sex, height, weight, chronic medical conditions, regular medication use, smoking status, sleep duration and nocturnal awakening frequency, caffeine-consumption frequency, exercise habits and frequency, and dietary habits. The analyzed participants reported no chronic medical conditions or regular medication use expected to substantially affect cardiovascular function and were therefore characterized as healthy young adults. The remaining questionnaire variables were collected to characterize the cohort and were not used as exclusion criteria. All participants provided written informed consent prior to participation. This study was approved by the Institutional Review Board (IRB) of Sangmyung University, Seoul, Republic of Korea (Approval No. SMUIRB(S-2024-013)). Each sample was classified and stored according to an experimental simulation condition that categorized alcohol consumption into four levels. Each data sample contains an rPPG signal (30 Hz) extracted from facial video and its corresponding cPPG signal (255 Hz). rPPG was extracted from facial video using a kernelized correlation filter (KCF)-based face tracker [15] combined with a modified chrominance-based (CHROM) method [16]. The retained preprocessing code applies a third-order Butterworth bandpass filter with zero-phase forward–backward filtering (filtfilt [17]); the archived CHROM routine also contains the De Haan chrominance bandpass step and an additional 0.7–4.0 Hz Butterworth bandpass stage. The rPPG signal served as input to the reconstruction model, whereas the cPPG signal served as the supervised reconstruction target and the post hoc reference. The duration of the archived paired cPPG cycle was also used to restore each reconstructed cycle to the paired 255 Hz time grid. Reference cPPG fiducial locations were not used to localize fiducial points in the reconstructed signals. To minimize confounding physiological factors and simplify the evaluation conditions, only data collected under the non-drinking condition were selected for use. Of these, 2 participants with missing data files were excluded, yielding a final dataset of 28 subjects, which was partitioned into training (20 subjects) and evaluation (8 subjects) sets on a subject-wise basis. In addition, the publicly available UBFC-rPPG dataset [18] was employed to further evaluate the adopted reconstruction pipeline. UBFC-rPPG is a benchmark dataset widely used in rPPG research, consisting of simultaneously acquired facial video and contact PPG signals from 42 participants. A subject-wise train/test split of 30/12 was applied, and the reconstruction model was trained and evaluated exclusively on the UBFC-rPPG dataset. This experiment was conducted independently of the self-collected dataset to evaluate the adopted reconstruction pipeline under a different subject population. To further examine external generalization, an additional cross-dataset experiment was performed in which the model trained on the self-collected dataset was directly applied to UBFC-rPPG without UBFC-specific retraining. This external experiment used 4187 UBFC cardiac cycles from 42 subjects.

### 3.2. Signal Reconstruction

This study employed the rPPG-to-cPPG reconstruction model proposed by Kim et al. [4]. Among the three architectures examined in the original study, the three-hidden-layer dense regression model was adopted without architectural modification. The contribution of the present study lies in evaluating the reconstructed signals using downstream cPPG-derived vascular indices rather than in proposing a new reconstruction architecture.

The implemented regressor followed a dense 30–16–16–16–30 architecture. It consisted of a 30-dimensional input layer, three hidden layers containing 16 units each with ELU activations, and a 30-dimensional output layer. Training used the Adam optimizer with a learning rate of $1\times 10^{-3}$, weight decay of $1\times 10^{-3}$, a batch size of 32, a maximum of 200 epochs, and early stopping with a patience of 20 epochs. The random seed for the original single-split experiment was fixed at 42.

The training objective combined waveform- and derivative-domain L1 losses as shown in Equation (1):

(1) $$L=L_{\mathrm{wave}}+0.5L_{\mathrm{first}}+0.2L_{\mathrm{second}},$$

where $L_{\mathrm{wave}}={∥\hat{y}-y∥}_{1}$, $L_{\mathrm{first}}={∥\Delta \hat{y}-\Delta y∥}_{1}$, and $L_{\mathrm{second}}={∥\Delta^{2}\hat{y}-\Delta^{2}y∥}_{1}$. Here, $\hat{y}$ and y denote the reconstructed and reference cPPG cycles, respectively, and Δ denotes the discrete first-difference operator. The derivative-domain terms were included to encourage preservation of waveform slopes and curvature in addition to pointwise amplitude similarity.

For model training, rPPG and cPPG signals acquired simultaneously during the same recording session were segmented into individual cardiac cycles and paired according to their temporal correspondence. Each cycle was independently normalized to the range [0,1] using min–max normalization and resampled to 30 points by cubic interpolation. The model received a 30-point rPPG cycle as input and generated the corresponding 30-point reconstructed waveform. Model training and evaluation used a subject-wise train–test split, and no participant was included in both subsets.

The 30-point representation was used only to standardize waveform length for model learning. After reconstruction, each cycle was mapped by cubic interpolation to the length of its paired cardiac-cycle segment before fiducial localization and timing-error calculation. The cycle duration used for this mapping was obtained from the pre-paired cardiac-cycle segment in the synchronized rPPG–cPPG dataset. Reference cPPG fiducial locations were not used to determine the reconstructed-signal $T_{s}$, notch, or $T_{d}$ locations.

A matched-ID audit of the retained analysis files found no duplicate subject–cycle identifiers among 4555 paired rPPG–cPPG cycles, confirmed one-to-one rPPG–cPPG pairing, and verified that every reconstructed evaluation cycle used the same reference cPPG cycle identifier as the paired raw rPPG cycle. Accordingly, the timing analysis should be interpreted as a paired-cycle morphology-preservation evaluation under known cycle boundaries rather than as a fully raw-only, continuous-video fiducial-detection pipeline.

Although an 800 ms cardiac cycle represented by 30 points has an average control-point spacing of approximately 27 ms, the cubic-interpolated waveform was evaluated on the paired 255 Hz grid, whose sample interval is approximately 3.92 ms. Consequently, the detected locations were not restricted to 27 ms increments. This finer evaluation grid does not imply recovery of physiological information lost during 30-point downsampling; the effect of resampling was therefore quantified separately through the round-trip interpolation analysis.

**Figure 1:**
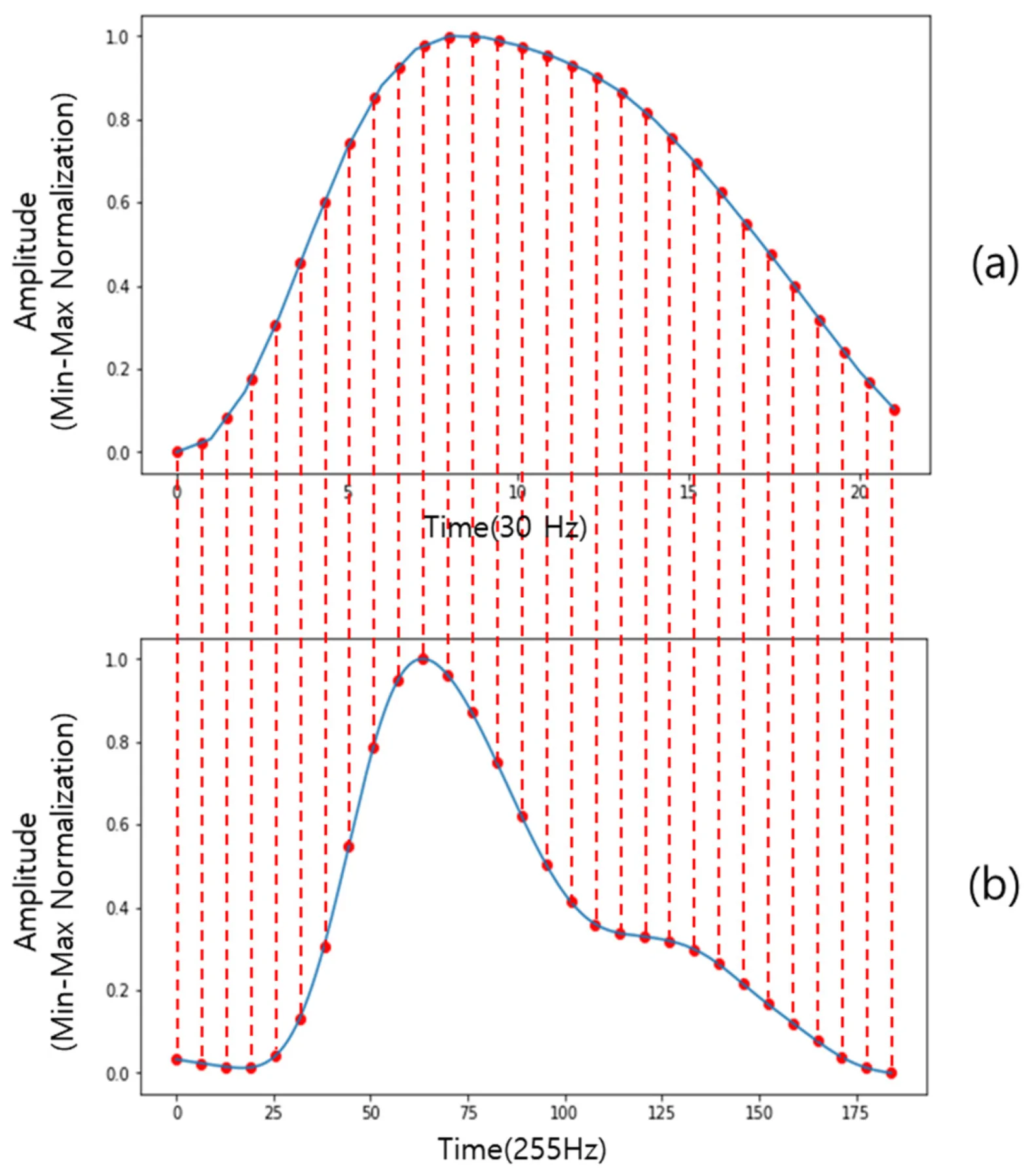
Example of cycle-by-cycle signal correspondence. (**a**) A single rPPG cycle acquired at 30 Hz; red dots indicate the original sampled points and the blue line indicates the interpolated waveform. (**b**) The corresponding cPPG cycle acquired at 255 Hz and normalized to [0,1]. The red dashed lines indicate cycle-wise point correspondence between the two signals [4].

After waveform reconstruction, vascular indices were independently extracted from the reconstructed signals and corresponding reference cPPG signals. Reference cPPG fiducial points were not used to select representative windows or to localize fiducial points in the reconstructed signals. Figure 1, reproduced from the original reconstruction study [4], illustrates the cycle-correspondence and preprocessing procedure adopted for model training.

### 3.3. SDPPG Feature Extraction

The second derivative photoplethysmography (SDPPG) was computed from the reconstructed signals to extract vascular morphological features. As SDPPG enhances subtle waveform variations in the original signal and renders the a, b, c, d, and e feature points more distinctly, it is widely employed in morphological analyses related to arterial elasticity and reflected wave characteristics [6,14]. In this study, each pulse waveform was first smoothed using a five-point moving average, after which the first and second derivatives were computed sequentially using numerical gradients. The resulting SDPPG signal was additionally smoothed using a three-point moving average before derivative-domain peak detection to mitigate high-frequency noise amplification. Because derivative-domain PPG morphology is sensitive to smoothing and filtering choices, identical processing parameters were applied to all signal types throughout the comparison [17]. Pulse-wave fiducial detection was performed independently for each reconstructed cycle: the systolic peak was identified as the dominant positive peak within the first 70% of the cycle, the dicrotic notch was searched as the minimum between 0.03 and 0.45 cycle lengths after the systolic peak, and the post-notch $T_{d}$ candidate was searched after the notch.

In the main computational analysis, $T_{d}$ was operationally defined as the post-notch location returned by the adopted detector. When a local maximum was present within the predefined post-notch search interval, that maximum was selected. If no local maximum was found, the maximum within the search interval was assigned as a fallback post-notch candidate. Therefore, the main analysis evaluates agreement under a consistent computational fiducial definition and does not assume that every returned $T_{d}$ corresponds to a physiologically distinct diastolic peak. A strict-local-peak sensitivity analysis, excluding all fallback assignments, was additionally performed. The strict-versus-fallback classification was conducted as a retrospective audit separately from the main index-extraction analysis. When the archived audit outputs did not permit an unambiguous cycle-level assignment, the corresponding status was reported as audit status unavailable. This category reflects a limitation of the retrospective audit and does not represent a third physiological waveform class or a detection failure.

The a feature point was defined as the largest positive extremum within the early portion of each SDPPG cycle when available, or otherwise as the largest positive extremum in the cycle. The b feature point was defined as the first local minimum following a, and the e feature point was estimated as the first local maximum after b using a minimum peak-distance threshold of 0.05L, where *L* denotes the cycle length. No prominence threshold was imposed in the adopted detector; because the sign of this e candidate was not constrained in the original detector, its positivity was also audited. The same detection rules were applied to the reference cPPG only after reconstructed-signal fiducial detection had been completed. In this study, the detector-returned a, b, and e candidates were used to compute the exploratory sAI. Their detection availability was distinguished from the accuracy of their derivative-domain amplitudes.

### 3.4. Morphological Validation Using Vascular Indices

In this study, cPPG-derived vascular indices were computed based on morphological feature points extracted from the reconstructed signals. These indices are used to quantitatively characterize the systolic and diastolic structure of the pulse waveform, the relative magnitude of the reflected wave, and properties related to arterial elasticity and vascular aging. Four indices were calculated in this study—SI, RI, sAI, and AIx—and each was computed in an identical manner for both the reconstructed signals and the reference cPPG.

In its conventional form, the Stiffness Index (SI) is a height-dependent timing surrogate related to pulse-wave velocity (PWV). Because the full aortic path length cannot be measured from a single PPG signal, participant height is commonly used as an approximation of pulse travel distance, and SI is calculated as $\mathrm{Height}/(T_{d}-T_{s})$, where $T_{s}$ denotes the systolic peak and $T_{d}$ conventionally denotes a secondary diastolic component.

Because the same participant height was used in both the reconstructed- signal and reference-cPPG SI calculations, between-participant height differences may inflate their apparent association. The height-independent delay $\Delta T=T_{d}-T_{s}$ was therefore evaluated separately to assess preservation of systolic-to-post-notch timing without the shared height term. In the main analysis of this study, however, $T_{d}$ denotes the detector-defined post-notch candidate, which may include a fallback assignment rather than a strict local diastolic peak. Accordingly, the reported SI should be interpreted as an operational timing-preservation measure within the paired-cycle evaluation rather than as an independently validated clinical measurement of arterial stiffness or PWV.

In its conventional form, the Reflection Index (RI) is defined as the ratio of the diastolic or secondary-wave amplitude ($P_{d}$) to the systolic peak amplitude ($P_{s}$). It is commonly interpreted as describing the relative magnitude of the secondary or reflected component of the peripheral pulse waveform. Earlier return of the reflected component with increasing arterial stiffness may alter the timing and amplitude of the secondary component, thereby affecting RI [8,19].

In the main analysis of this study, $P_{d}$ was obtained at the detector-defined post-notch $T_{d}$ location and could therefore correspond to either a strict local peak or a fallback segment maximum. Accordingly, fallback-inclusive RI should be interpreted as an operational post-notch-to-systolic amplitude ratio under the adopted detector rather than as a measurement calculated exclusively from a physiologically distinct reflected-wave peak.

The conventional Aging Index (AI) is an SDPPG-based index that reflects vascular aging using the amplitudes of the characteristic SDPPG feature points. In previous studies, AI has commonly been defined using all five SDPPG feature points (*a*, *b*, *c*, *d*, and *e*) [6]. However, in reconstructed signals, the *c* and *d* feature points were not consistently detected due to the limited waveform morphology and the sensitivity of higher-order derivatives [20]. Therefore, this study adopted a sAI formulation based only on the *a*, *b*, and *e* feature points, following previous studies that employed simplified SDPPG-derived aging indices when consistent detection of the *c* and *d* feature points was difficult [21]. Previous studies have also reported that the relative amplitudes of individual SDPPG feature points are associated with vascular aging and arterial characteristics [7]. The sAI used in this study is not mathematically equivalent to the conventional five-point SDPPG Aging Index. Therefore, its values should be interpreted as a morphology-derived surrogate measure for comparative evaluation within this study and should not be directly compared with conventional AI reference ranges.

In conventional pulse-wave analysis, the Augmentation Index (AIx) describes the contribution of a reflected or secondary component to the pulse waveform. In this study, AIx was calculated as

$$\mathrm{AIx}=\frac{P_{d}-P_{s}}{P_{s}-P_{o}}\times 100,$$

where $P_{o}$, $P_{s}$, and $P_{d}$ denote the pulse-onset, systolic-peak, and post-notch candidate amplitudes, respectively. Because each cardiac cycle was min–max normalized, $P_{o}=0$ and $P_{s}=1$ under the adopted formulation. Consequently,

AIx=100(RI−1),

and AIx is mathematically a linear transformation of RI rather than an independent index in the present analysis.

Furthermore, $P_{d}$ may correspond to a fallback post-notch candidate rather than a strict local diastolic peak. The reported AIx should therefore be interpreted as an operational RI-derived secondary measure within this study and should not be treated as a conventional pressure-wave augmentation index or compared directly with its clinical reference ranges.

Table 1 summarizes the mathematical definitions and physiological interpretations of the cPPG-derived vascular indices used in this study. The morphological validity of the reconstructed signals was assessed by comparing the vascular indices derived from the reconstructed signals with those obtained from the corresponding reference cPPG. Raw rPPG-derived indices were also calculated when the same fiducial-detection criteria were satisfied, enabling a direct raw-versus-reconstructed comparison on the subset of cycles for which raw rPPG fiducials were detectable. For cycles in which the required raw rPPG fiducial points could not be detected, the contribution of reconstruction was interpreted as increasing the number of analyzable cycles rather than necessarily improving every index value on already analyzable raw cycles.

Unless otherwise stated, the main SI, ΔT, RI, and AIx analyses were fallback-inclusive. These indices were based on the detector-defined post-notch candidate and should not be interpreted as indices calculated exclusively from physiologically distinct diastolic peaks. Results restricted to strict post-notch local maxima are reported separately as a sensitivity analysis.

### 3.5. Representative Window Selection

To identify a representative pulse-wave segment, overlapping candidate windows of 10 s duration were generated for each subject with a step size of 5 s (50% overlap). Within each candidate window, the systolic peak, dicrotic notch, and detector-defined post-notch $T_{d}$ candidate were detected from each reconstructed cardiac cycle. The position of each fiducial point was normalized by the corresponding cycle length as $p_{k,i}=\frac{l_{k,i}}{L_{i}}$, where $l_{k,i}$ denotes the sample index of fiducial point *k* in the *i*-th cardiac cycle and $L_{i}$ is the corresponding cycle length. The cycle-to-cycle variability of each fiducial point was quantified using the standard deviation of the normalized positions. Fiducial instability was then defined as $I_{\mathrm{fid}}=\frac{\sigma_{\mathrm{sys}}+\sigma_{\mathrm{notch}}+\sigma_{\mathrm{dia}}}{3}$, where $\sigma_{\mathrm{sys}}$, $\sigma_{\mathrm{notch}}$, and $\sigma_{\mathrm{dia}}$ denote the standard deviations of the normalized systolic peak, dicrotic notch, and detector-defined post-notch candidate positions, respectively. Candidate windows were ranked by fiducial detectability, ascending $I_{\mathrm{fid}}$, and reconstructed-signal SSQI. The SSQI threshold was set to 0.41 following the criterion adopted in PPGFeat [22]. Windows with SSQI values above this threshold were regarded as having acceptable waveform quality for morphological analysis. Candidate windows containing fewer than three complete cardiac cycles were excluded from further analysis. Reference cPPG signals were not used during window selection and were accessed only after representative window selection for quantitative evaluation.

### 3.6. Statistical Analysis

Pearson’s correlation coefficient was used to evaluate the linear association between vascular indices derived from the reconstructed signals and the corresponding reference cPPG measurements. Agreement was additionally assessed using the intraclass correlation coefficient calculated with a two-way random-effects model for single measurements and absolute agreement (ICC(2,1)), together with Bland–Altman analysis. For Bland–Altman analysis, the difference was calculated as reconstructed minus reference values, and the bias was defined as the mean difference. The 95% limits of agreement were calculated as the bias ±1.96× the standard deviation of the differences. Because multiple cardiac cycles were extracted from each participant, subject-level median values were used as the primary agreement analysis, while cycle-level results were interpreted as supplementary analyses describing within-recording variability. For the original eight-participant single-split analysis, confidence intervals for subject-level Pearson correlation coefficients were calculated using the Fisher *z* transformation, whereas confidence intervals for MAE, bias, and ICC(2,1) were estimated using 5000 participant-level bootstrap resamples with a fixed random seed (20260911). For the pooled held-out predictions from LOSO cross-validation, the confidence intervals reported in Section 4.8 were obtained by resampling participant IDs 5000 times and recalculating each statistic, including Pearson *r*, within every bootstrap replicate. Metric-specific paired bootstrap analyses for raw-versus-reconstructed comparisons likewise used 5000 participant-level resamples with the same fixed seed. The strict-local-peak sensitivity analysis was treated separately as a descriptive cycle-level audit. Because this subset comprised only 37 repeated cycles from four participants, inferential confidence intervals were not reported for its correlation or ICC estimates. The corresponding MAE, bias, limits of agreement, correlation, and ICC were interpreted only as descriptive characteristics of the retained subset and not as population-level estimates.

### 3.7. Robustness and Subject-Wise Cross-Validation Experiments

Three additional experiments were performed to evaluate robustness to input signal quality, sensitivity to the preprocessing bandwidth, and subject-level generalization. All three reuse the fiducial-detection and vascular-index code of the main evaluation without modification, so their results are directly comparable with the analyses reported above.

**SNR-based noise injection**. Additive white Gaussian noise was added to the input rPPG cycles after mapping to the paired 255 Hz cycle grid at signal-to-noise ratios (SNR) of 20, 15, 10, 5, and 0 dB, together with a clean condition. Noise was injected after per-cycle min–max normalization and before the 30-point cubic resampling, because that representation is the earliest stage of the retained pipeline that still carries the temporal resolution of the input rPPG; injecting the noise after resampling would bypass the implicit averaging of the resampling step and overstate the degradation. The reference cPPG cycles were never modified and the reconstruction model was not retrained, so the experiment characterizes the robustness of the deployed pipeline rather than of a noise-adapted model. Ten independent noise realizations were drawn at each SNR level using a fixed base seed (20260912); all reported values are means across realizations, and the standard deviation across realizations is used to verify that no conclusion depends on a particular noise draw.

**Bandpass upper-cutoff sensitivity**. The influence of the upper bandpass cutoff was examined by additionally low-pass filtering the input rPPG cycles at 3.0, 2.5, 2.0, and 1.5 Hz using a zero-phase third-order Butterworth filter. The filter family, order, and zero-phase implementation were kept identical to the baseline preprocessing so that the cutoff was the only independent variable. Because a single cardiac cycle is shorter than the period associated with the lowest cutoff tested, each cycle was periodically extended by tiling it seven times before filtering, and the center segment was retained; the operation therefore corresponds to removing harmonics above the cutoff in the harmonic basis of the cycle itself. Cutoffs wider than the 42–240 bpm baseline could not be evaluated. A spectral audit of every retained rPPG artifact showed that spectral content above 4 Hz had already been removed at signal-extraction time, and the offline chrominance-extraction and rPPG–cPPG cycle-synchronization procedures that would be required to re-derive the signals from the raw recordings under a wider cutoff were no longer available; re-deriving the region of interest with a reconstructed tracker would have changed the extraction method itself and confounded the cutoff variable. The spectral composition of the input rPPG and of the reference cPPG was therefore quantified directly, in order to establish how much spectral content the preprocessing band excludes.

**Subject-wise cross-validation**. Leave-one-subject-out (LOSO) cross-validation was performed over all 28 participants in the self-collected dataset, and subject-wise five-fold cross-validation was performed as an independent cross-check. Both experiments used the same network architecture, loss formulation, optimizer, learning rate, weight decay, batch size, epoch budget, and early-stopping patience as the original single-split experiment described in Section 3.2. The cross-validation seed was fixed at 20260912.

Within each outer fold, the early-stopping validation set was constructed exclusively from participants in the training partition. Four training participants were selected for validation, and no model-selection decision was informed by the held-out evaluation participant or participants. Subject independence was enforced throughout; cardiac cycles from the same participant were never divided across the training, validation, and evaluation partitions. Because min–max normalization was applied independently to each cycle, no normalization statistics were shared across partitions. Fold assignments, participant lists, and per-fold results were recorded for all 28 LOSO folds and 5 subject-wise cross-validation folds.

## 4. Results

### 4.1. Raw-Versus-Reconstructed Candidate Availability and Index Accuracy

**Figure 2:**
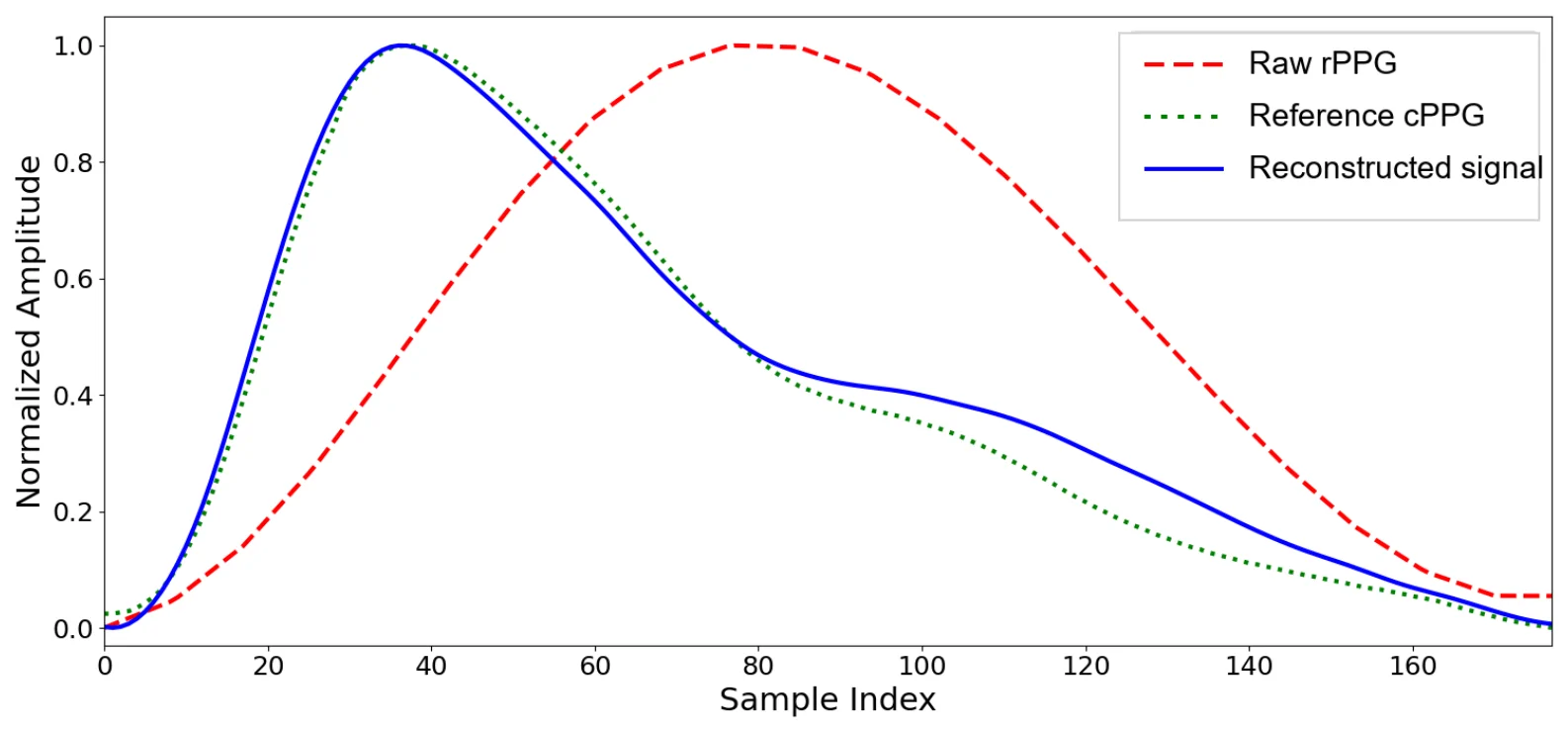
Comparison of raw rPPG, the reconstructed signal, and reference cPPG. The raw rPPG signal preserves pulse periodicity but provides less distinct morphology for joint pulse-wave and SDPPG candidate extraction, whereas the reconstructed signal provides clearer candidate structures under the adopted detector.

To investigate whether raw rPPG signals can directly support vascular index estimation, the waveforms of the raw rPPG, reconstructed signals, and reference cPPG were first compared. As shown in Figure 2, although the raw rPPG signal preserves the overall periodicity of the pulse waveform, it exhibits a considerably smoother morphology than the corresponding cPPG signal. In particular, characteristic vascular morphological components, including the dicrotic notch and diastolic peak, are substantially attenuated or indistinct in the raw rPPG waveform. In contrast, the reconstructed signal more closely resembles the morphology of the reference cPPG and provides clearer candidate structures for the adopted detector. These observations suggest that raw rPPG provides lower joint availability of the pulse-wave and SDPPG candidates required by the adopted detector. However, metric-specific analyses showed that larger raw-rPPG subsets remained analyzable for indices requiring fewer fiducial points; reconstruction was therefore evaluated in terms of both candidate availability and index accuracy. To quantify this, fiducial point detection was performed on both the raw rPPG and reconstructed signals using the same 1378 cardiac cycles from the subject-wise test set (Table 2). A cardiac cycle was considered successfully detected only if the systolic peak, dicrotic notch, and detector-defined post-notch $T_{d}$ candidate were identified in the pulse waveform and the corresponding a, b, and e points were detected in the expected temporal order in the SDPPG waveform. Detection succeeded for 23.37% of the raw rPPG cycles but for all reconstructed cycles under the adopted detector, indicating that waveform reconstruction increases the number of analyzable cycles while still requiring detector-dependence audits for reflected-wave landmarks.

For the subset of cycles in which raw rPPG fiducials were detectable by the same analysis code, raw rPPG, reconstructed signals, and reference cPPG were compared on matched cycles. The matched subset was defined separately for each index using only the fiducial points required for that index. For example, ΔT and SI required the systolic and diastolic candidates, whereas sAI required the SDPPG a, b, and e points. As summarized in Table 3, the reconstructed signal did not materially improve the fallback-inclusive ΔT error compared with raw rPPG on this already-detectable subset. However, reconstruction substantially reduced errors for RI, sAI, and the RI-derived AIx. These results indicate that the main benefit of reconstruction is twofold: it increases the number of analyzable cycles and improves some amplitude-related indices under the adopted detector, while timing accuracy on high-quality raw-detectable cycles remains similar.

Subject-wise paired bootstrap analysis showed that the raw-minus-reconstructed MAE difference was close to zero for ΔT (−0.19 ms; 95% CI, −0.49 to 0.05 ms) and slightly favored raw rPPG for SI (−0.0069 m/s; 95% CI, −0.0140 to −0.0011 m/s). In contrast, the difference favored reconstruction for RI (0.134; 95% CI, 0.085 to 0.190), sAI (0.400; 95% CI, 0.222 to 0.576), and AIx (13.41%; 95% CI, 8.37 to 18.95%). Positive values indicate lower MAE for the reconstructed signal. Therefore, the timing-related results should be interpreted as comparable fallback-inclusive timing behavior rather than accuracy improvement or general recovery of true local diastolic timing, whereas reflected-wave amplitude indices showed clearer improvement after reconstruction.

To further evaluate the cycle-coverage benefit, cycles in which raw rPPG index estimation failed but reconstructed-signal and reference-cPPG estimates were available were analyzed separately (Table 4). This analysis indicates that reconstruction provided additional analyzable cycles beyond the raw-detectable subset.

**Figure 3:**
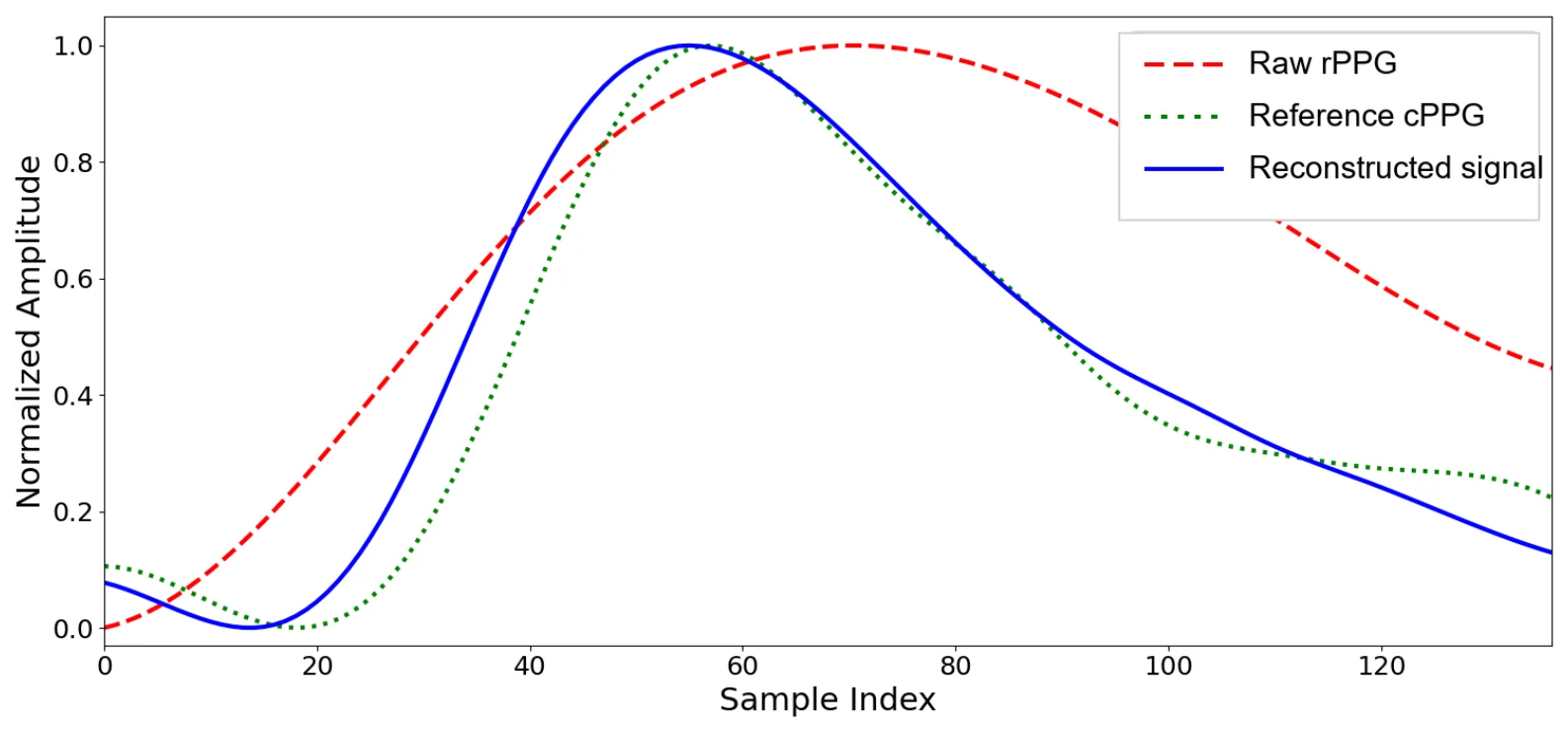
Representative waveform reconstruction results on the UBFC-rPPG dataset, demonstrating improved waveform-level similarity under subject-independent evaluation on the UBFC-rPPG dataset.

Furthermore, the reconstruction model was evaluated on the UBFC-rPPG dataset [18] using a subject-wise train/test split of 30/12. Across 1201 cardiac cycles extracted from 12 held-out subjects, the reconstructed signals achieved a mean Pearson correlation coefficient (PCC) of 0.926 and a mean cosine similarity of 0.974, as illustrated in Figure 3 and summarized in Table 5. These results indicate that the reconstruction model improves waveform-level similarity under subject-independent evaluation on the UBFC-rPPG dataset. Whether this waveform similarity is sufficient for local reflected-wave landmark recovery and vascular index estimation is investigated in the following sections.

Although the reconstruction model achieved high waveform similarity, these metrics alone do not suggest whether the reconstructed signals preserve the physiological morphology required for vascular index estimation. Therefore, the following analyses investigate whether the reconstructed signals retain the fiducial information necessary for SDPPG-based vascular index computation.

### 4.2. Morphological Reconstruction Increases Fiducial-Candidate Availability

**Figure 4:**
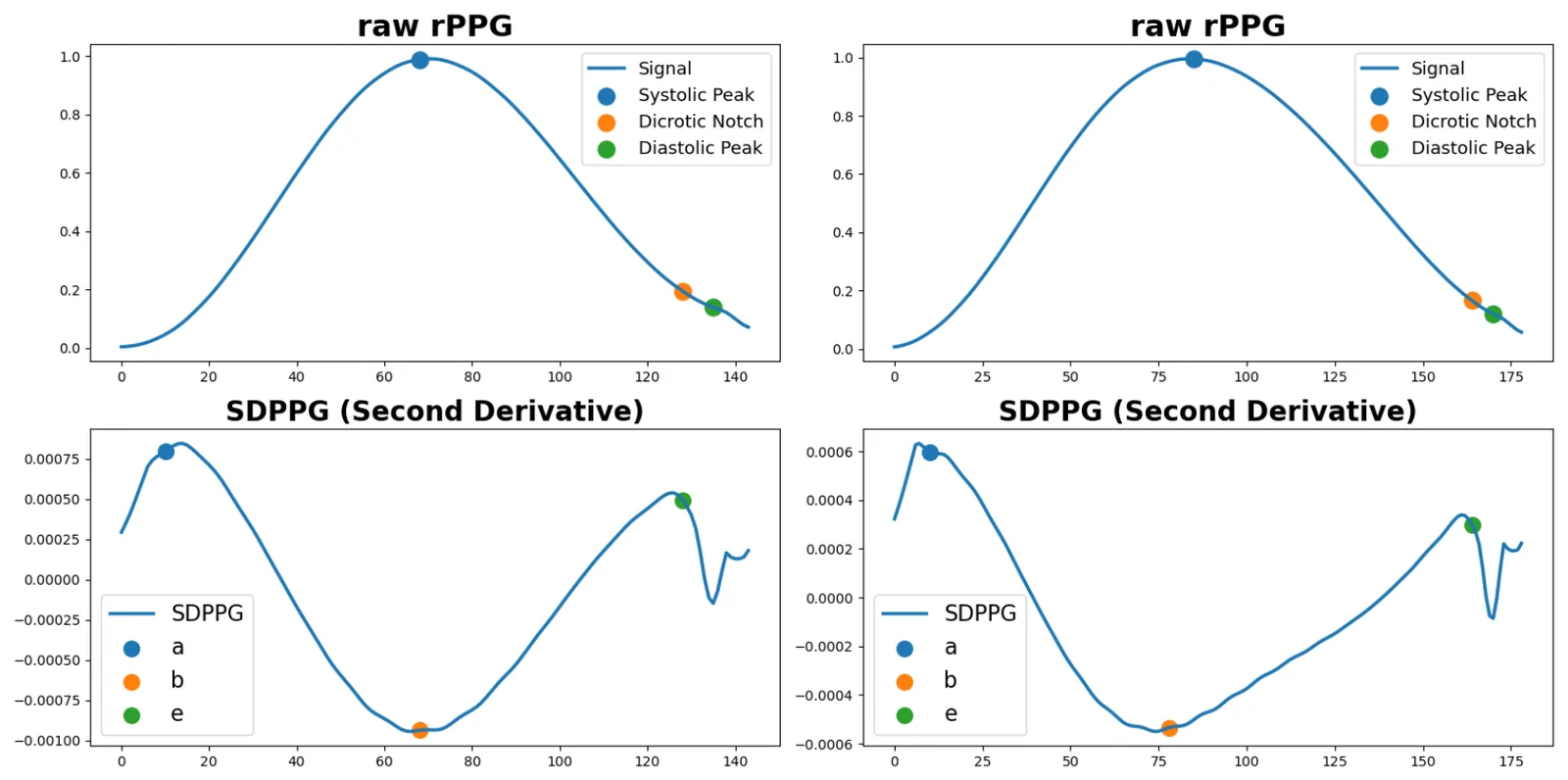
Representative examples illustrating fiducial point detection from raw rPPG cycles and their corresponding second derivative photoplethysmography (SDPPG) waveforms. In each raw rPPG waveform, the marked points indicate, from left to right, the systolic peak, dicrotic notch, and detector-defined post-notch $T_{d}$ candidate. In each SDPPG waveform, the marked points indicate, from left to right, the a, b, and e points. Although the raw rPPG cycles exhibit smooth and generally similar pulse-wave shapes, the locations of the systolic peak, dicrotic notch, and detector-defined post-notch $T_{d}$ candidate vary across cardiac cycles, and their corresponding SDPPG waveforms show substantial morphological variability with inconsistently localized a, b, and e feature points. These examples illustrate the instability of both pulse-wave fiducial point detection and derivative-based feature-point localization in raw rPPG signals, resulting in the difficulty of directly computing SDPPG-based vascular indices.

**Figure 5:**
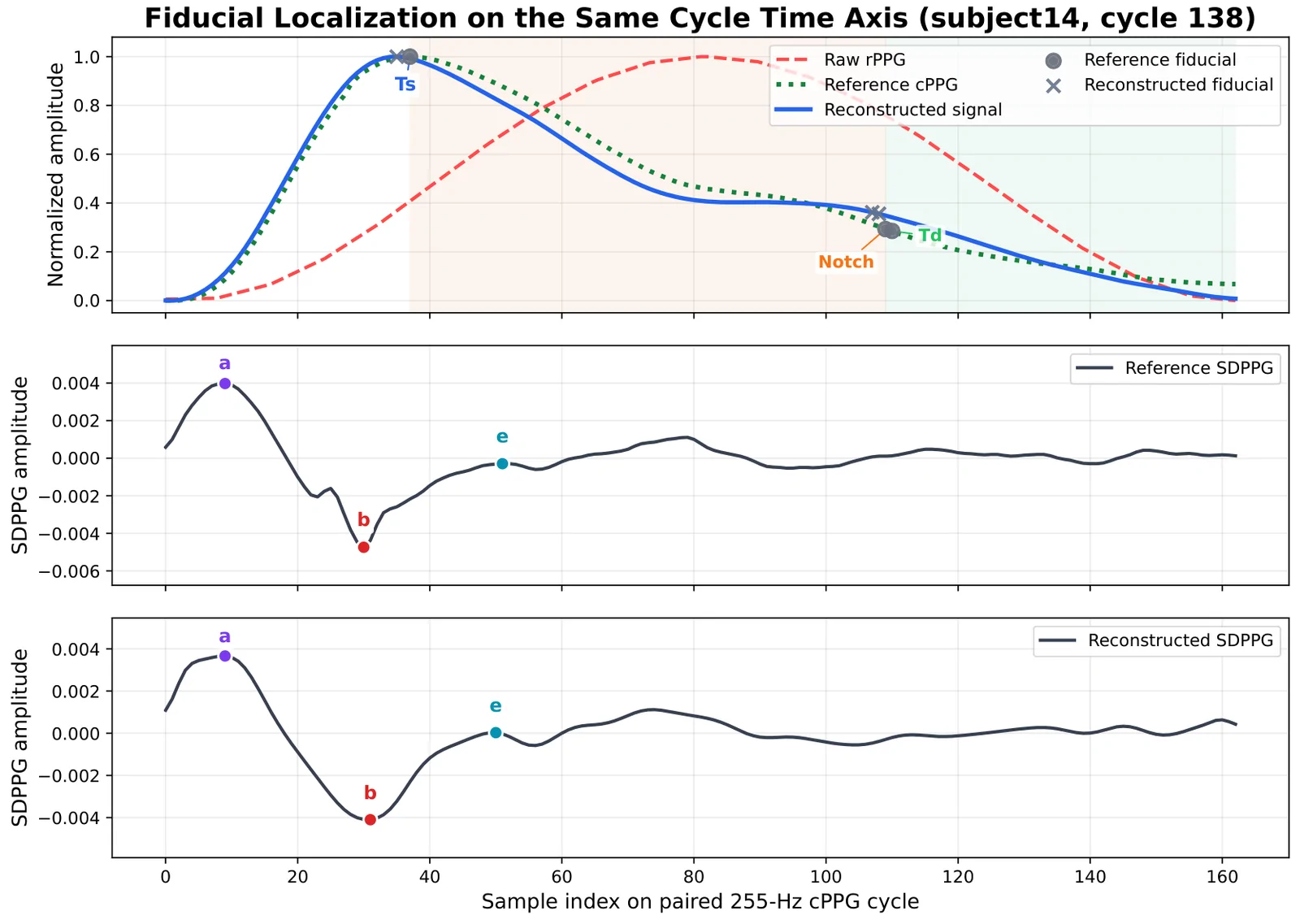
Rechecked visualization of fiducial point localization on a representative cycle using a common paired-cycle time axis. The upper panel shows the raw rPPG, reference cPPG, and reconstructed signal after cycle-wise normalization. The detected systolic peak ($T_{s}$), dicrotic notch, and detector-defined post-notch $T_{d}$ candidate are marked for the reference and reconstructed signals, and the notch and diastolic search ranges are shaded. The middle and lower panels show the corresponding reference and reconstructed SDPPG waveforms with the detected a, b, and e points.

Figure 4 shows that, although raw rPPG cycles exhibit relatively similar pulse-wave shapes, their corresponding SDPPG waveforms show substantial beat-to-beat variability, resulting in inconsistent localization of the pulse-wave and SDPPG fiducial points. In contrast, the reconstructed signal in Figure 5 more closely resembles the reference cPPG and enables the adopted detector to return the pulse-wave and SDPPG candidates required for index calculation. Candidate availability does not, however, establish that every returned location corresponds to a physiologically distinct fiducial point. These qualitative results indicate that waveform reconstruction provides more detector-usable structure than raw rPPG, while the detector audit below evaluates whether the diastolic candidate represents a local peak or a fallback selection.

The detector used for Figure 5 and in the quantitative analyses was audited to determine whether the marked points were supported by local extrema or by fallback rules (Table 6). The audit was performed on the 1075 reconstructed–reference cycle pairs that passed the reference-cPPG validity criteria; this represents a conditional paired subset of the 1378 reconstructed cycles evaluated for overall candidate-extraction coverage in Table 2. In reference cPPG, 332 of 1075 cycles (30.88%) were classified as containing a strict post-notch local peak, and 572 cycles (53.21%) were classified as fallback segment-maximum assignments. The remaining 171 cycles (15.91%) contained a detector-returned post-notch candidate but could not be classified unambiguously from the archived retrospective-audit outputs and were therefore reported as audit status unavailable. No specific waveform or algorithmic cause was assigned because the underlying reason was not independently verified cycle by cycle. No cycles were classified as detection failures. In reconstructed cycles, $T_{d}$ was identified as a local peak in 5.02% of cycles and as a post-notch segment-maximum fallback candidate in 94.98% of cycles, with the fallback candidate lying on a descending segment in 1021 cycles. The main detector returned an SDPPG *e* candidate for all 1075 reconstructed cycles in this paired subset. Polarity-audit status was available for 1074 cycles (99.91%), and 69.18% of the auditable *e* candidates were positive maxima. The unavailable polarity-audit status for one cycle does not indicate failure of the candidate returned by the main index-computation pipeline. These audit results show that the fallback-inclusive vascular-index analyses should be interpreted as detector-dependent cycle-coverage analyses under the adopted detector, rather than as evidence that a true local diastolic peak was recovered in most reconstructed cycles.

Strict local $T_{d}$ peaks were identified in 54 of 1075 reconstructed cycles (5.02%; six participants) and in 332 of 1075 paired reference cPPG cycles (30.88%; seven participants). Both signals contained a strict local $T_{d}$ in only 37 matched cycles from four participants. For raw rPPG, strict local peaks were found in 5 of 1378 cycles (0.36%; three participants), and only 2 cycles from two participants had strict peaks in both raw rPPG and reference cPPG. Consequently, strict raw-versus-reference agreement statistics were not calculated.

### 4.3. Strict-Local-Peak Sensitivity Analysis

An exploratory sensitivity analysis was additionally performed using only cycles in which both the reconstructed signal and paired reference cPPG contained a strict post-notch local maximum. This criterion retained 37 matched cycles from four participants. The results are summarized in Table 7.

Within this restricted subset, the descriptive cycle-level estimates for ΔT were r=0.622 and ICC(2,1) =0.322, with an MAE of 31.48 ms and wide limits of agreement. SI showed a higher descriptive correlation, although the same participant height was included in both SI calculations. RI and the algebraically equivalent AIx showed negligible agreement. Because the 37 cycles represented repeated observations from only four participants, these statistics characterize only the retained strict-local-peak subset. They were not treated as independent observations or used for population-level or subject-level inference.

### 4.4. Fallback-Inclusive Fiducial and Interpolation Analysis

To quantitatively evaluate the detector-dependent preservation of pulse-wave morphology, the temporal and amplitude differences of the detected fiducial points were analyzed under the fallback-inclusive detector. As summarized in Table 8, the systolic peak exhibited the smallest temporal error (18.42 ms), whereas the dicrotic notch (35.12 ms) and post-notch $T_{d}$ candidate (34.18 ms) showed relatively larger but comparable timing errors. Considering that a typical cardiac cycle under resting conditions spans approximately 800–1000 ms, these temporal errors correspond to only about 2–4% of a single cardiac cycle; however, the detector audit above indicates that the diastolic-peak timing result depends heavily on the fallback rule and should not be interpreted as broad recovery of true local $T_{d}$ peaks. Table 9 summarizes the amplitude errors of the detected fiducial points. Since each cardiac cycle was normalized to the range [0, 1] before waveform reconstruction, the reported amplitude errors directly represent normalized waveform deviations. The systolic peak exhibited a negligible amplitude error (0.0004), whereas relatively larger errors were observed for the dicrotic notch (0.0729) and post-notch candidate (0.0857), corresponding to approximately 7–9% of the normalized waveform amplitude. Overall, systolic timing was more robust than reflected-wave amplitude and derivative-domain morphology, whereas diastolic timing requires conservative interpretation because many $T_{d}$ candidates were fallback selections.

Because the reconstruction model used 30-point cycle representations, the effect of downsampling and restoring cPPG cycles was additionally quantified by applying a 30-point round-trip interpolation to reference cPPG cycles and rerunning the same fiducial detector. As summarized in Table 10, 30-point round-trip interpolation preserved detection for all ΔT, SI, RI, and AIx cycles and introduced a median ΔT error of 0 ms, with a 95th-percentile absolute error of 7.84 ms. Individual fiducial point errors were also small for $T_{s}$ and the notch and largest for $T_{d}$ (Table 11), indicating that the main round-trip timing uncertainty arises from the post-notch diastolic candidate rather than from the systolic peak. The error was larger for the derivative-amplitude-based sAI, supporting the observation that SDPPG amplitude indices are more sensitive to interpolation and derivative processing than timing-based indices.

### 4.5. Quantitative Evaluation of cPPG-Derived Vascular Indices

As summarized in Table 12, the reconstructed signals showed high waveform similarity with the reference cPPG. Vascular indices were subsequently evaluated using both all valid cardiac cycles and representative analysis windows.

The adopted reconstruction pipeline was additionally evaluated on the UBFC-rPPG dataset. Because subject height information was unavailable, SI was excluded from the analysis. Quantitative results for all valid cycles and representative analysis windows are summarized in Table 13 and Table 14, respectively.

To emulate practical vascular assessment, representative windows were selected for each subject using the fiducial-stability-based strategy described in Section 3.5 and compared with the corresponding reference cPPG (Table 14). Because only the selected stable cycles were used rather than all valid cycles, the reported reference cPPG mean values differ from those in Table 13.

An additional external cross-dataset analysis was conducted by applying the model trained on the self-collected dataset directly to UBFC-rPPG without UBFC-specific retraining. As summarized in Table 15, this setting was substantially more challenging than the within-UBFC train/test evaluation. The adopted detector returned the required candidates for all reconstructed cycles, including fallback post-notch assignments, and the detector-defined ΔT showed a positive cycle-level association with the reference-cPPG-derived values. However, waveform similarity was markedly lower than in the within-dataset UBFC evaluation, and RI, AIx, and sAI showed little or no agreement. Therefore, the external results indicate that some detector-defined timing correspondence was retained under dataset shift, whereas amplitude- and derivative-domain vascular-index agreement did not generalize robustly.

The self-collected dataset was evaluated using both all valid cardiac cycles and representative analysis windows (Table 16 and Table 17). Representative window selection reduced the MAE for SI, RI, and AIx, whereas the sAI MAE slightly increased. The largest improvement was observed for SI.

**Figure 6:**
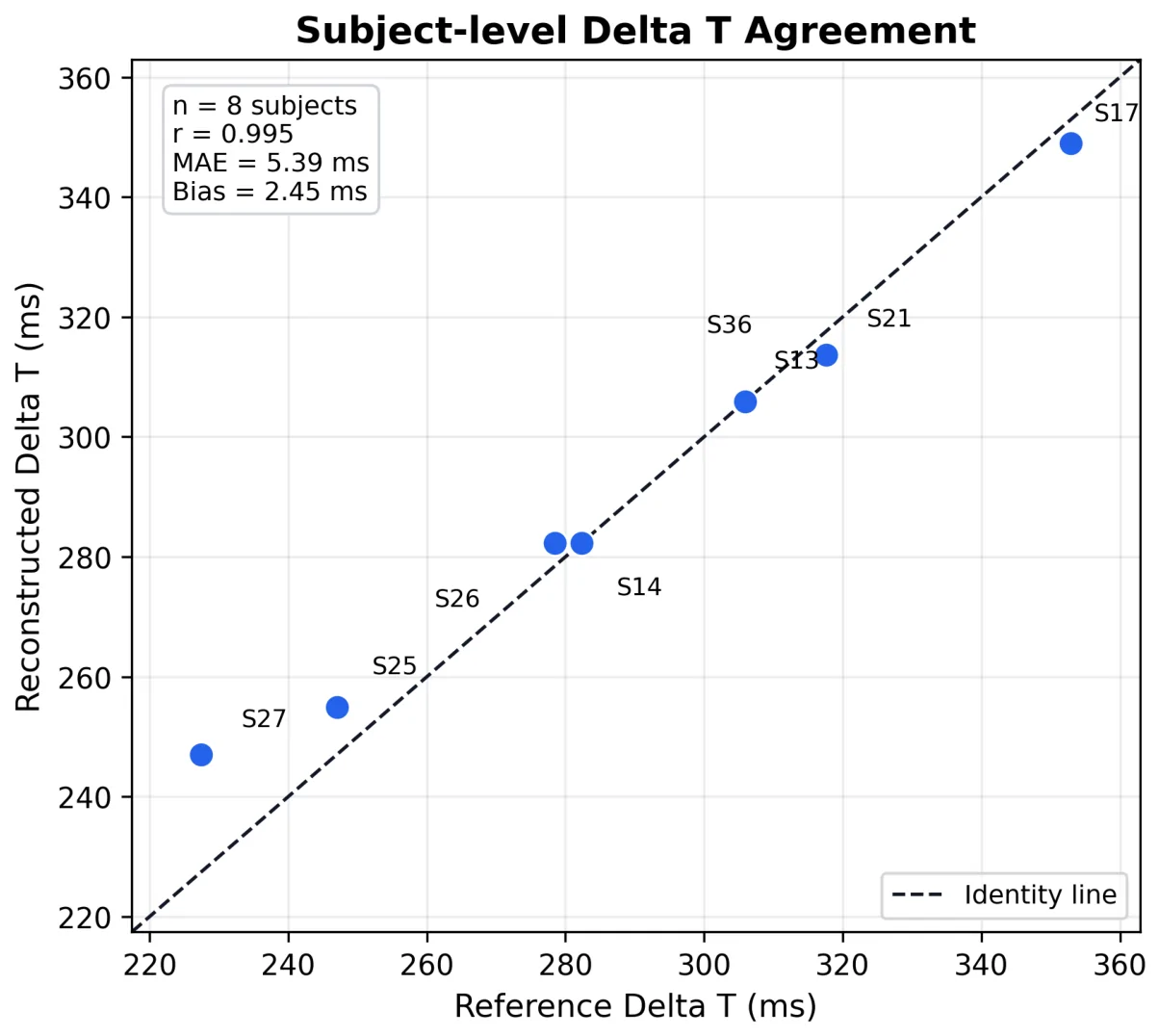
Subject-level scatter plot of the height-independent, detector-defined systolic-to-post-notch delay ΔT. Each point represents one participant’s median reference ΔT and reconstructed-signal-derived ΔT across valid cardiac cycles. The dashed line indicates the identity line.

Because SI includes subject height, the height-independent, detector-defined systolic-to-post-notch delay ΔT was additionally analyzed. As summarized in Table 18 and visualized in Figure 6, the fallback-inclusive ΔT analysis showed strong subject-level agreement, with a Pearson correlation coefficient of 0.995 and an ICC(2,1) of 0.977. Confidence intervals were added to quantify uncertainty arising from the small number of evaluation participants. In light of the detector audit, this result is interpreted as detector-dependent evidence that the paired-cycle fallback-inclusive pipeline preserves a detector-defined systolic-to-post-notch timing surrogate, while the SI value itself should not be interpreted as a direct clinical stiffness measurement or as proof that true local diastolic peaks were recovered in most cycles.

To complement the cycle-level evaluation, a subject-level agreement analysis was performed by calculating the median value of all valid cardiac cycles for each participant. The median was selected instead of the mean because it is more robust to occasional outlier cycles caused by fiducial point detection errors and physiological beat-to-beat variability. As summarized in Table 19, SI showed the strongest agreement between the reconstructed and reference cPPG measurements, whereas RI and AIx exhibited relatively weak agreement. Because AIx was calculated as a linear transformation of RI under the adopted formulation, the two indices yielded identical Pearson correlation coefficients and ICC values despite different bias and limits of agreement.

**Figure 7:**
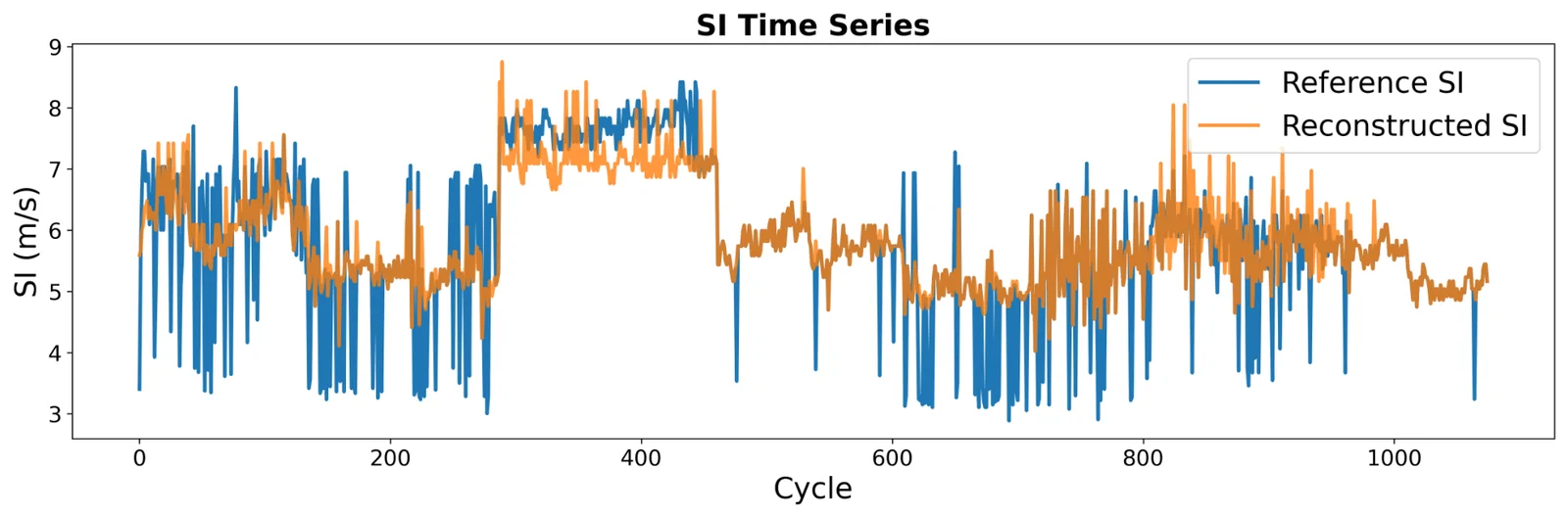
Time-series comparison of the Stiffness Index (SI) computed from the reference cPPG and reconstructed signals. Although the reconstructed SI closely follows the temporal trend of the reference cPPG measurements for most cardiac cycles, a limited number of outlier cycles exhibit relatively larger deviations, contributing disproportionately to the overall standard deviation.

**Figure 8:**
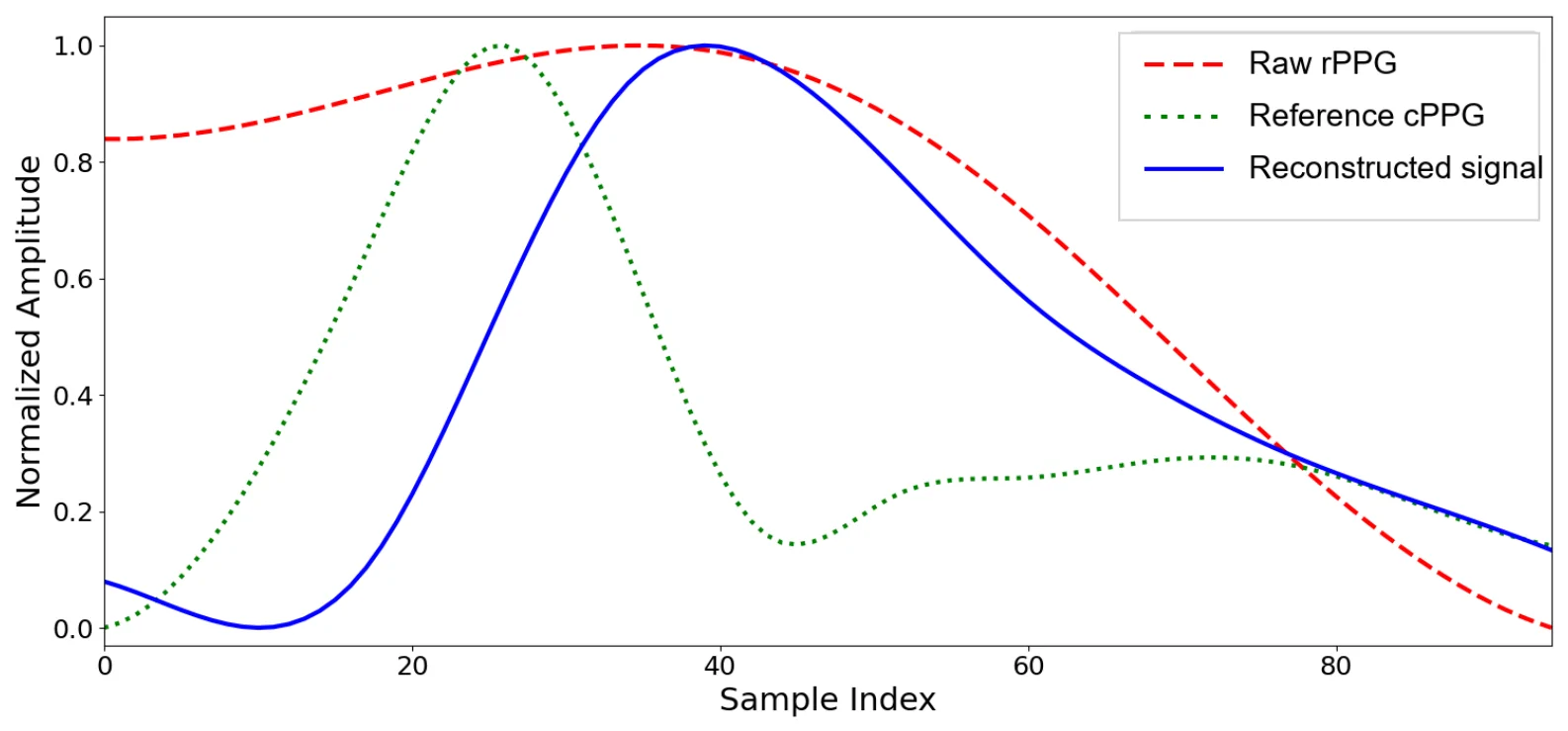
Representative outlier cardiac cycle corresponding to one of the large SI deviations observed in Figure 7. In this example, degraded morphology in the input rPPG coincides with less accurate reconstruction and fiducial localization. This example illustrates a possible contribution of input quality but does not establish the cause of all outlier cycles.

Relatively large standard deviations nevertheless remained for several cPPG-derived vascular indices. To investigate this, the SI values extracted from representative cardiac cycles were visualized as a time series (Figure 7). The reconstructed SI closely followed the temporal trend of the reference cPPG for most cycles, while a limited number of cycles exhibited noticeably larger deviations that contributed disproportionately to the reported standard deviation. One such outlier cycle was examined further (Figure 8): the input rPPG exhibited degraded pulse-wave morphology, coinciding with less accurate reconstruction and errors in fiducial point localization. Some large-error cycles coincided with visibly degraded input rPPG, suggesting that poor input quality may be one contributor. However, the causes of all outlier cycles were not independently classified, and input signal quality should be regarded as one plausible factor among others affecting the robustness of vascular index estimation.

**Figure 9:**
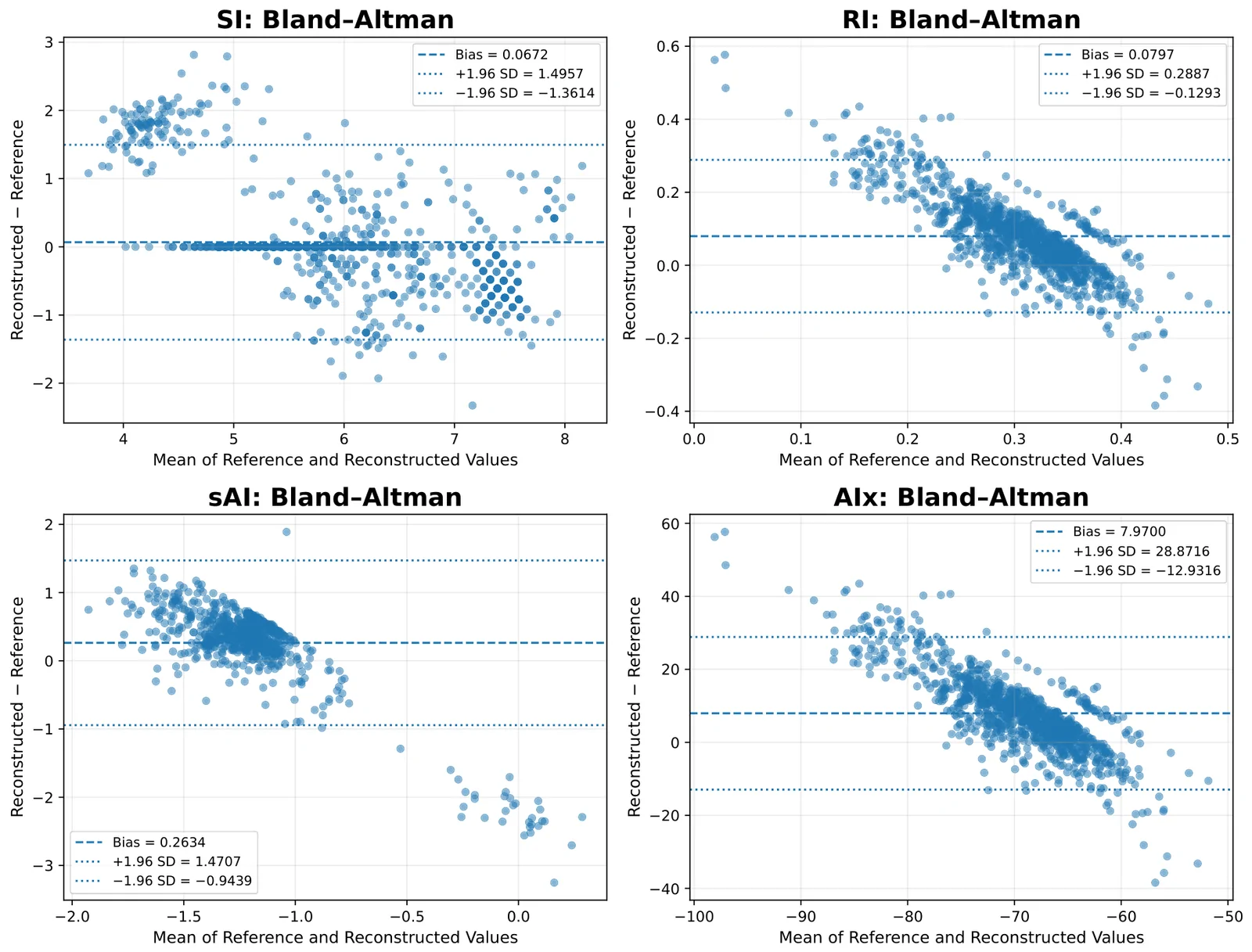
Cycle-level Bland–Altman analysis comparing the vascular indices derived from the reconstructed signals with those obtained from the corresponding reference cPPG.

Cycle-level agreement between the reconstructed-signal and reference cPPG indices was further evaluated by Bland–Altman analysis (Figure 9), which demonstrated index-dependent agreement, with relatively small bias for SI and larger disagreement for the amplitude- and derivative-dependent indices, particularly the sAI and AIx.

The corresponding Pearson correlation coefficients, ICC(2,1), bias, and 95% limits of agreement are given in Table 20. SI showed the highest agreement, exhibiting a Pearson correlation coefficient of r=0.805, an ICC(2,1) of 0.744, and a small systematic bias. In contrast, RI and AIx showed relatively weak agreement. The sAI showed no meaningful cycle-level agreement, with a Pearson correlation coefficient of −0.026 and an ICC(2,1) of −0.012, indicating that the current reconstruction framework did not adequately preserve the SDPPG amplitude relationships required for reliable sAI estimation.

To further investigate the poor sAI agreement, the individual SDPPG amplitude ratios used in the simplified formulation were analyzed. As shown in Table 21, neither b/a nor e/a showed meaningful agreement between the reconstructed signals and reference cPPG. The component-level MAE and bias further indicate that the poor sAI result was not caused only by the final sAI formula, but by insufficient preservation of derivative-domain amplitude relationships.

### 4.6. Robustness to Input rPPG Signal Quality

The effect of degraded input quality was quantified by injecting additive white Gaussian noise into the input rPPG at controlled SNR levels (Table 22). Candidate extraction on the reconstructed signals remained at 100% under the adopted fallback-inclusive detector in every condition, so degradation manifested as localization and index error rather than as detection failure. Waveform similarity decreased only moderately, from a Pearson correlation of 0.945 in the clean condition to 0.918 at 0 dB. Fiducial localization degraded very unevenly: the systolic peak error was essentially unchanged (18.05 ms clean versus 19.39 ms at 0 dB), whereas the dicrotic notch error nearly doubled (35.38 ms versus 68.68 ms) and the diastolic candidate error increased from 35.31 ms to 50.89 ms. Across the ten noise realizations, the standard deviation was at most 0.0013 for waveform correlation and 1.7 ms for notch localization, indicating that these trends are not artifacts of a particular noise draw.

**Figure 10:**
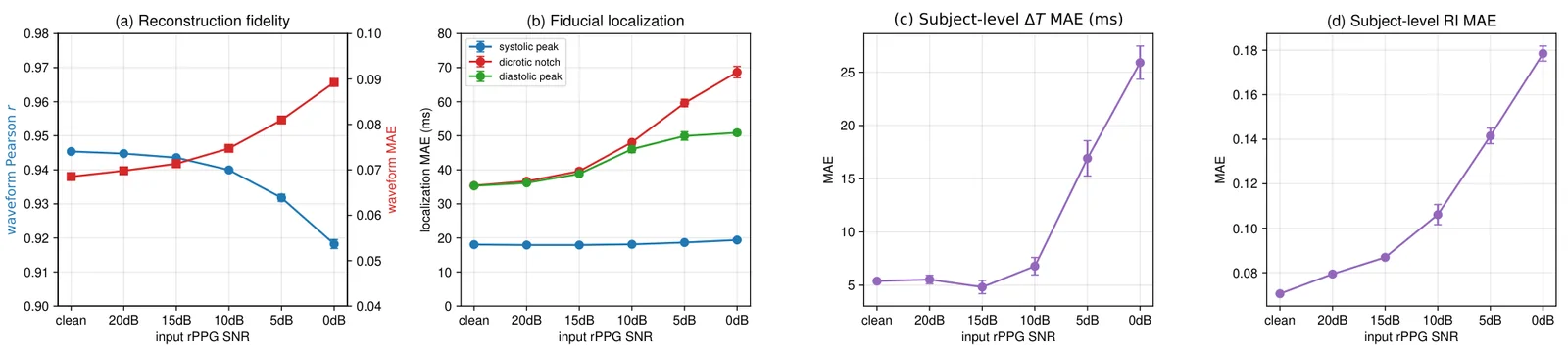
Effect of input rPPG signal quality on the reconstruction pipeline. Additive white Gaussian noise was applied to the input rPPG at the signal-to-noise ratios shown; markers are means over ten independent noise realizations and error bars indicate the standard deviation across realizations. (**a**) Waveform fidelity of the reconstructed signal against the reference cPPG. (**b**) Fiducial localization error: the systolic peak is almost unaffected across the tested range, whereas the dicrotic notch error nearly doubles. (**c**) Subject-level error of the height-independent, detector-defined systolic-to-post-notch delay ΔT. (**d**) Subject-level error of the reflected-wave index RI. The axes in panels (**a**,**b**) use the same ranges as the corresponding panels of Figure 11, so that the magnitude of the noise effect and of the cutoff effect can be compared directly.

At the subject level the vascular indices showed a two-regime response (Table 23), and the overall degradation pattern is summarized in Figure 10. The fallback-inclusive timing-related measures were relatively stable down to approximately 10 dB and then degraded sharply: the ΔT MAE remained between 4.83 and 6.79 ms from the clean condition to 10 dB but rose to 16.91 ms at 5 dB and 25.91 ms at 0 dB, and the SI MAE followed the same pattern. Notably, the subject-level Pearson correlation for ΔT and SI remained above 0.98 at every SNR level, whereas the corresponding ICC(2,1) fell from 0.977 to 0.778 for ΔT and from 0.959 to 0.833 for SI. Noise therefore left the between-subject ranking of the fallback-inclusive timing measures largely intact while progressively destroying their absolute agreement, which is consistent with the growing systematic bias observed at low SNR. The reflected-wave index RI degraded monotonically from an MAE of 0.071 to 0.179 with its ICC(2,1) falling from 0.176 to 0.018, and the number of cycles in which the SDPPG *a*, *b*, and *e* points could all be detected decreased from 721 to 644. The sAI error did not increase with noise, but this reflects the absence of any agreement in the clean condition rather than robustness. At the cycle level the ΔT MAE doubled from 25.72 ms to 51.19 ms and the correlation fell from 0.695 to 0.425.

**Figure 11:**
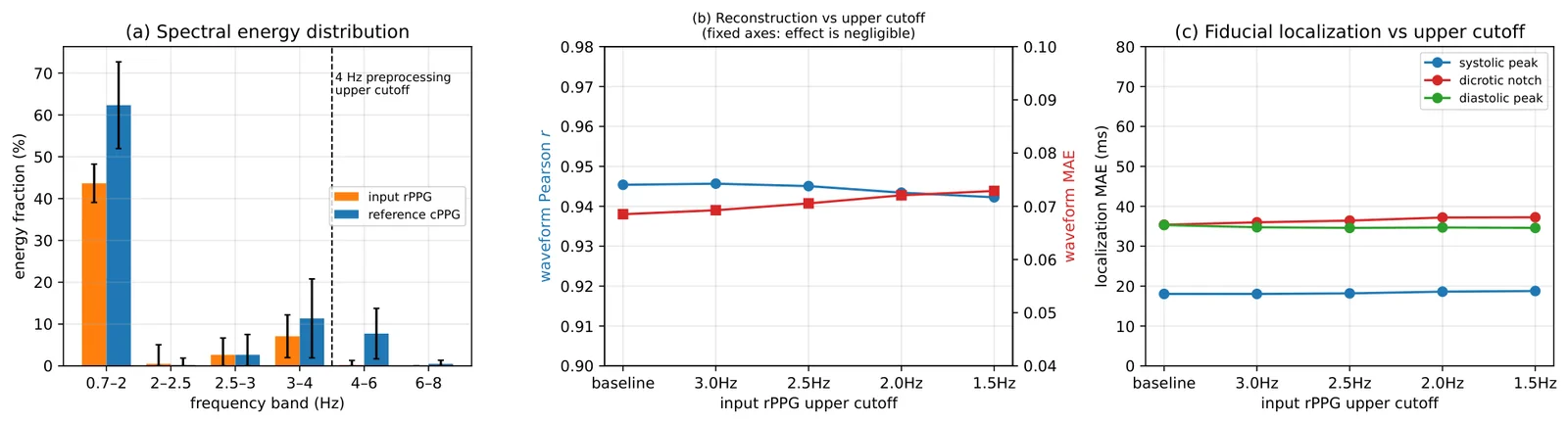
Spectral composition of the evaluation cycles and sensitivity of the pipeline to the upper cutoff of the input rPPG. (**a**) Mean fraction of total cycle energy per frequency band for the input rPPG and for the simultaneously acquired reference cPPG; the dashed line marks the 4 Hz upper edge of the preprocessing band. The reference cPPG retains 7.73% of its energy between 4 and 6 Hz, where the input rPPG retains only 0.19%; error bars show the standard deviation across cycles and their lower whisker is clipped at zero because an energy fraction cannot be negative. (**b**) Reconstruction fidelity and (**c**) fiducial localization error as the upper cutoff of the input rPPG is narrowed. The vertical axes in (**b**,**c**) are fixed to the same ranges as Figure 10 rather than auto-scaled, because the changes across the whole sweep are on the order of 0.003 in correlation and 2 ms in notch localization, and an auto-scaled axis would visually exaggerate them.

### 4.7. Sensitivity to the Bandpass Upper Cutoff

**Figure 12:**
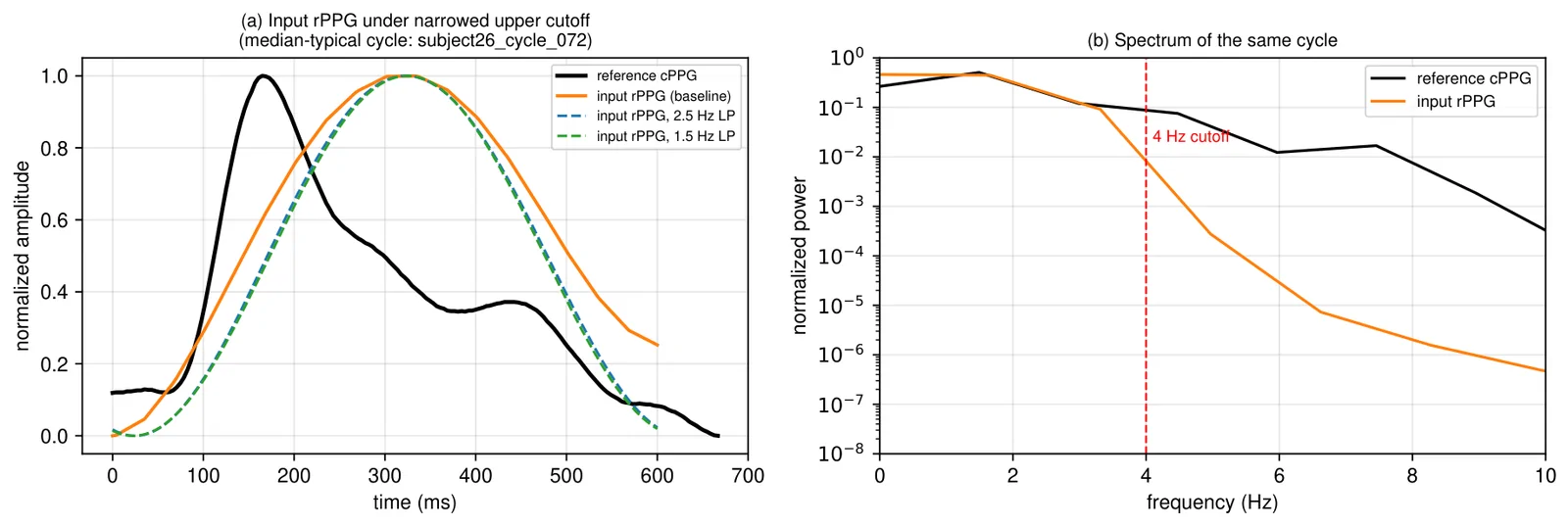
Representative single cycle illustrating why only the narrowing direction could be evaluated. The cycle was not hand-picked: for every evaluation cycle the absolute difference between the systolic-peak times of the input rPPG and of the paired reference cPPG was computed, and the cycle at the median of that distribution is shown, so the example is typical rather than a best or a worst case. (**a**) The input rPPG cycle and its paired reference cPPG cycle, together with the same input cycle after additional zero-phase low-pass filtering at 2.5 and 1.5 Hz; the two filtered traces lie almost on top of the unfiltered input. The visible offset between the input rPPG and reference cPPG systolic peaks is typical of this paired dataset (median 168.6 ms across 1075 cycles) and reflects the peripheral pulse transit delay between the facial and finger measurement sites together with independent per-signal cycle segmentation; it does not affect the indices analyzed here, which are all computed within a single signal. (**b**) Normalized power spectra of the same pair of cycles; the dashed line marks the 4 Hz preprocessing cutoff. The reference cPPG retains appreciable power above 4 Hz whereas the input rPPG does not, so a wider upper cutoff cannot be evaluated from the retained signals.

The spectral audit of the evaluation cycles is summarized in the upper part of Table 24 and in Figure 11a, with a representative single cycle shown in Figure 12. The input rPPG retained only 0.19% of its cycle energy between 4 and 6 Hz and 0.001% between 6 and 8 Hz, whereas the simultaneously acquired reference cPPG retained 7.73% and 0.51% in the same bands. The 42–240 bpm preprocessing band therefore excludes a spectral region that the contact reference demonstrably occupies, showing that the retained preprocessing band excluded spectral content that was present in the reference cPPG and could be relevant to reflected-wave morphology. However, because this content is absent from every retained rPPG artifact, a wider-cutoff condition could not be evaluated, and only narrower cutoffs were testable.

Narrowing the upper cutoff produced relatively small changes in waveform similarity and detector-defined timing measures, whereas RI agreement decreased progressively (lower part of Table 24; Figure 11b,c). Reducing the cutoff from the 4 Hz baseline to 1.5 Hz changed the waveform correlation from 0.9454 to 0.9423 and the waveform MAE from 0.0685 to 0.0729, and the dicrotic notch localization error increased only from 35.38 ms to 37.25 ms. The subject-level ΔT and SI errors were unchanged, and the only index that responded systematically was RI, whose cycle-level correlation with the reference declined from 0.235 to 0.140 and whose ICC(2,1) declined from 0.089 to 0.024. Fiducial detection performed directly on the input rPPG increased with narrower cutoffs, from 91.44% to 95.72%, which reflects the smoothing-induced tendency of the detector to accept a notch candidate rather than improved morphology. Because a wider cutoff could not be evaluated, these results characterize sensitivity to additional removal of components already retained below 4 Hz and do not determine whether preserving frequencies above 4 Hz would improve reflected-wave recovery.

The input pulse-morphology candidate rate reported in this analysis requires only the pulse-domain systolic, notch, and post-notch candidates and is therefore not directly comparable with the 23.37% six-candidate rate in Table 2, which additionally requires the SDPPG a, b, and e candidates.

### 4.8. Subject-Wise Cross-Validation

Leave-one-subject-out and subject-wise five-fold cross-validation produced nearly identical estimates for the timing-related indices, whereas the amplitude- and derivative-dependent indices remained weak and showed greater variation between validation schemes (Table 25).

Across all 28 subjects, the subject-level ΔT MAE was 15.55 ms under LOSO and 15.62 ms under five-fold validation, with Pearson correlations of 0.909 and 0.908 and ICC(2,1) values of 0.837 in both analyses. SI showed the same pattern, with MAEs of 0.213 and 0.214 m/s and ICC(2,1) values of 0.926 in both analyses. The amplitude- and derivative-dependent indices remained weak: under LOSO, RI had a correlation of 0.354 (95% CI, −0.028 to 0.687) and an ICC(2,1) of 0.139 (95% CI, −0.011 to 0.250), and sAI had a correlation of 0.198 (95% CI, −0.184 to 0.538) and an ICC(2,1) of 0.060. Under LOSO validation, the confidence intervals for both the correlation and ICC of RI included zero, and the corresponding sAI estimates were also compatible with no agreement. The five-fold point estimates were similarly weak or negative. Reconstructed-signal candidate extraction remained at 100% under the fallback-inclusive detector in every fold.

**Figure 13:**
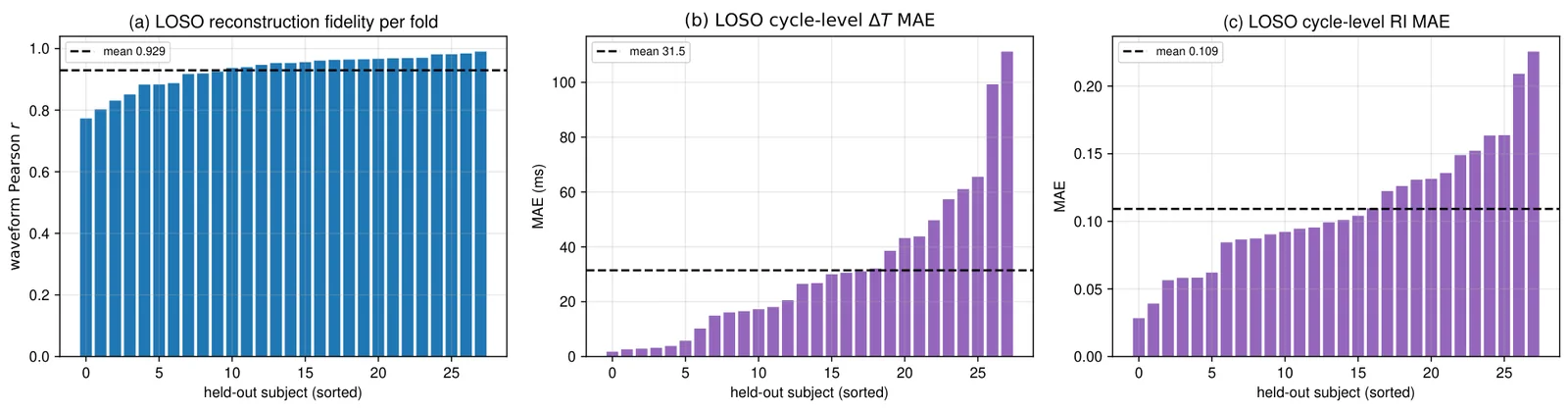
Per-fold results of leave-one-subject-out cross-validation over the 28 subjects of the self-collected dataset. Each bar is one held-out subject, and folds are sorted by the plotted quantity. (**a**) Mean cycle-level waveform correlation, (**b**) mean cycle-level ΔT error, and (**c**) mean cycle-level RI error; dashed lines mark the across-fold means. The spread is wide, with waveform correlation ranging from 0.773 to 0.990 and ΔT error from 1.77 ms to 111.23 ms, indicating that the cross-validated averages conceal substantial between-subject differences.

Per-fold variation was substantial (Figure 13). Across the 28 LOSO folds the mean cycle-level waveform correlation for a held-out subject ranged from 0.773 to 0.990, and the mean cycle-level ΔT error ranged from 1.77 ms to 111.23 ms. The cross-validated estimates should therefore be read as cohort averages that conceal large between-subject differences, and the more favorable single-split values should be attributed in part to the particular eight subjects that were held out.

## 5. Discussion

The present study investigated whether cPPG-derived vascular indices can be estimated from reconstructed rPPG signals. Rather than treating waveform similarity as sufficient evidence, this study examined fiducial localization and downstream vascular-index agreement. The results indicate that reconstruction increased the number of cycles in which pulse-wave and SDPPG fiducials could be detected. However, the matched-cycle raw rPPG comparison showed that reconstruction should not be interpreted as uniformly improving every index on cycles that were already analyzable in the raw signal. Its clearest benefit was the expansion of analyzable cycles, with additional error reductions for RI and the RI-derived AIx on the matched subset of cycles that was already analyzable in raw rPPG.

Among the evaluated indices, SI exhibited the strongest subject-level agreement with the corresponding reference-cPPG-derived values. However, the same participant height was included in both SI calculations, and the post-notch location used in the main analysis frequently originated from the detector’s fallback rule. The height-independent ΔT analysis showed strong subject-level agreement under this operational fiducial definition, but this should be interpreted as reproducibility of a detector-defined systolic-to-post-notch interval under known paired-cycle boundaries, rather than as independent validation of arterial stiffness, PWV, or consistent recovery of a distinct physiological diastolic peak.

In the exploratory strict-local-peak analysis (Table 7), only 37 matched cycles from four participants were available. Within this restricted subset, the descriptive cycle-level estimates suggested moderate correlation but limited absolute agreement for ΔT, whereas RI and AIx showed negligible agreement. The strict analysis was therefore insufficient for population-level validation but confirmed that candidate availability and physiological peak recovery should not be treated as equivalent.

The ΔT limits of agreement were substantially wider at the cycle level than at the subject-median level. This difference is expected because individual-cycle estimates are more sensitive to beat-to-beat waveform variability, input rPPG quality, and occasional fiducial localization errors, whereas subject-level medians suppress isolated outlier cycles. Therefore, the cycle-level analysis was retained as a supplementary robustness check rather than the primary evidence for subject-level agreement.

RI and AIx showed only weak agreement with reference cPPG. This is consistent with the larger amplitude errors observed at the dicrotic notch and diastolic peak, which directly affect reflected-wave indices. In addition, AIx was mathematically a linear transformation of RI under the adopted normalized formulation; therefore, it should be interpreted as a secondary RI-derived measure rather than as an independent vascular biomarker in this study.

The sAI did not show meaningful subject-level or cycle-level agreement. The component analysis of b/a and e/a further indicated that the derivative-domain amplitude ratios themselves were not preserved. This suggests that the poor sAI agreement was not merely a limitation of the simplified formula, but reflected insufficient preservation of SDPPG amplitude relationships by the current reconstruction framework. Thus, the present model appears more suitable for fallback-inclusive timing-surrogate analysis than for derivative-amplitude-based aging-index estimation, but it does not yet provide robust recovery of local diastolic morphology.

The representative-window analysis improved several indices by selecting windows with stable detector-returned reconstructed-signal candidates and acceptable SSQI. Importantly, reference cPPG was not used to select these windows; it was used only after selection for quantitative evaluation. This design reduces the risk that representative-window results were inflated by selecting windows according to reference agreement.

The noise-injection experiment clarified which parts of the analysis depend on input signal quality. Systolic-peak localization was almost unaffected by noise, while the dicrotic notch error nearly doubled between the clean condition and 0 dB. Under the specific additive-white-Gaussian-noise conditions examined, a transition in error behavior was observed near 10 dB: the fallback-inclusive timing indices changed relatively little at higher SNR levels but degraded more rapidly below this level. A dissociation also emerged at the subject level, where the Pearson correlation of ΔT and SI remained above 0.98 at every SNR while their ICC(2,1) fell substantially. Noise therefore left the between-subject ordering of the fallback-inclusive timing measures largely intact but progressively destroyed their absolute calibration, which means that correlation alone would have given an over-optimistic picture of robustness. These findings also provide additional quantitative context for the large-error outlier cycle illustrated in Figure 8, for which visibly degraded input rPPG was noted as one plausible, though not independently confirmed, contributor.

The spectral audit showed that the reference cPPG retained 7.73% of its cycle energy between 4 and 6 Hz, whereas the stored input rPPG retained only 0.19% in this band. Further narrowing the retained upper cutoff from 4.0 to 1.5 Hz produced only small changes in waveform similarity and detector-defined timing indices, although RI agreement decreased progressively. These results characterize the sensitivity of the current pipeline to additional removal of frequencies already present below 4 Hz. They do not establish that the original 4-Hz cutoff was optimal, that frequencies above 4 Hz are unnecessary, or that wider-band input would not improve reflected-wave recovery, because frequencies above 4 Hz were unavailable in the retained input signals.

Subject-wise cross-validation confirmed the qualitative conclusions of the study while providing a full-cohort estimate. Across all 28 subjects, timing-related agreement remained the strongest result. Under LOSO validation, the confidence intervals for the amplitude- and derivative-dependent indices included zero, while the corresponding five-fold point estimates were likewise weak or negative. The per-fold spread observed under LOSO, where the mean cycle-level ΔT error ranged from 1.77 ms to 111.23 ms across held-out subjects, was substantial. Because the cross-validated estimates average over every subject in the dataset, they should be regarded as the more reliable indication of subject-independent performance.

Several limitations should be acknowledged. First, the detector audit showed that reconstructed-cycle $T_{d}$ selection relied heavily on fallback post-notch candidates; consequently, fallback-inclusive ΔT, SI, RI, and AIx results should be treated as detector-dependent sensitivity analyses rather than as validation that local diastolic morphology was generally recovered. Second, the external UBFC-rPPG experiment showed that the self-collected-dataset-trained model did not fully generalize waveform morphology or amplitude-dependent vascular indices across datasets. Although reconstructed-signal candidate availability under the adopted detector and fallback-inclusive ΔT agreement were retained, RI, AIx, and sAI agreement remained weak under dataset shift. This indicates that broader cross-dataset training and validation are still required before the framework can be considered robust across camera, population, and acquisition-condition differences. Third, the bandpass cutoff analysis could only be performed in the narrowing direction. Spectral content above 4 Hz had already been removed from every retained rPPG signal at extraction time, and the offline chrominance-extraction and rPPG–cPPG cycle-synchronization procedures required to regenerate the signals from the raw recordings under a wider band were no longer available, so conditions such as 0.7–6 or 0.7–8 Hz could not be evaluated. Because the reference cPPG carries measurable energy in the 4–6 Hz band that the input rPPG does not, whether a wider acquisition bandwidth combined with a reconstruction model able to exploit it would improve reflected-wave recovery remains an open question; answering it requires re-extraction from the raw video with a preserved and reproducible preprocessing chain. Fourth, the noise-injection experiment used additive white Gaussian noise applied to the extracted rPPG cycles, which does not reproduce the structured disturbances of real recordings such as subject motion, illumination change, or face-tracking failure; the transition observed near 10 dB should therefore be interpreted only within the specific synthetic-noise conditions examined and not as a deployment threshold. Finally, although the validation now covers all 28 subjects of the self-collected dataset through leave-one-subject-out and subject-wise five-fold cross-validation, the cohort remains small and consists of healthy young adults recorded under controlled conditions, and the per-fold variation was large. Future studies should evaluate larger and more diverse populations, including older individuals and subjects with known vascular disease, compare multiple reconstruction models, and incorporate signal-quality-aware reconstruction and validation strategies.

## 6. Conclusions

This study evaluated the technical feasibility of using cPPG-derived vascular indices as downstream measures of morphology preservation in reconstructed rPPG signals. Reconstruction increased the availability of detector-defined pulse-wave and SDPPG candidates and enabled index calculation for more cycles than raw rPPG. Under the adopted computational fiducial definition, timing-related measures, including SI and the height-independent ΔT, showed more favorable agreement than amplitude- and derivative-dependent indices. RI and the RI-derived AIx showed limited agreement, while sAI showed no meaningful agreement.

The detector audit showed, however, that strict post-notch local maxima were present in only 5.02% of reconstructed cycles and that most reconstructed post-notch locations were fallback assignments. Thus, computational candidate availability did not guarantee recovery of a physiologically distinct diastolic peak. The exploratory strict analysis was limited to 37 matched cycles from four participants and showed moderate timing correlation but limited absolute agreement.

Accordingly, the present findings support the use of vascular-index calculations as a technical downstream framework for evaluating reconstructed rPPG morphology. They should not be interpreted as clinical validation of vascular status or as evidence that standalone non-contact vascular assessment has been established. Further studies require physiologically validated reflected-wave localization, independent cycle-boundary estimation, larger and more diverse cohorts, and direct clinical vascular reference measurements. 

## Figures and Tables

**Table 1:** Definition of cPPG-derived vascular indices used in this study.

Index	Formula	Physiological Meaning
SI (m/s)	$\frac{\mathrm{Height}}{T_{d}-T_{s}}$	Height-dependent, detector-defined systolic-to-post-notch timing measure.
ΔT (ms)	$T_{d}-T_{s}$	Height-independent, detector-defined systolic-to-post-notch time delay.
RI	$\frac{P_{d}}{P_{s}}$	Operational post-notch-to-systolic amplitude ratio under the adopted detector.
sAI	$\frac{b-e}{a}$	Exploratory SDPPG-derived index computed from the detector-returned *a*, *b*, and *e* candidates.
AIx (%)	$\frac{P_{d}-P_{s}}{P_{s}-P_{o}}\times 100$	RI-derived secondary measure under the adopted normalized formulation.

Abbreviations: $P_{s}$, systolic peak amplitude; $P_{d}$, post-notch candidate amplitude; $P_{o}$, pulse onset; $T_{s}$, time of the systolic peak; $T_{d}$, time of the detector-defined post-notch candidate.

**Table 2:** Candidate-extraction performance of the adopted pulse-wave and SDPPG detector for raw rPPG and reconstructed signals. Successful extraction required all detector-returned candidates used for index calculation and included fallback post-notch assignments.

Signal	Detected	Discarded	Candidate-Extraction Rate (%)
Raw rPPG	322	1056	23.37
Reconstructed signal	1378	0	100.00

**Table 3:** Metric-specific matched-cycle comparison of raw rPPG-derived and reconstructed-signal-derived indices against reference cPPG. The matched subset was defined separately for each index according to the fiducial points required for that index.

Index	Subjects	Cycles	Raw MAE	Raw *r*	Recon. MAE	Recon. *r*
ΔT (ms)	8	985	24.42	0.715	24.63	0.710
SI (m/s)	8	985	0.385	0.836	0.393	0.820
RI	8	985	0.245	−0.074	0.099	0.268
sAI	7	627	0.907	−0.086	0.500	−0.034
AIx (%)	8	985	24.46	−0.074	9.91	0.268

**Table 4:** Performance on cycles in which raw rPPG index estimation failed but reconstructed-signal and reference-cPPG estimates were available.

Index	Subjects	Cycles	Recon. MAE	Recon. *r*
ΔT (ms)	7	90	37.47	0.553
SI (m/s)	7	90	0.606	0.675
RI	7	90	0.130	0.021
sAI	6	94	0.521	−0.024
AIx (%)	7	90	12.98	0.021

**Table 5:** Reconstruction performance on the UBFC dataset (test: 12 subjects, 1201 cycles).

Metric	Mean	Std
PCC	0.926	0.079
Cosine Similarity	0.974	0.028

**Table 6:** Detector audit for reference and reconstructed cycles used in the self-collected evaluation. A strict $T_{d}$ required a post-notch local maximum, whereas fallback indicates that the maximum within the post-notch search interval was returned because no local maximum was found. Audit status unavailable indicates that the archived audit outputs did not permit an unambiguous retrospective classification. The audit was performed on the 1075 reconstructed–reference cycle pairs that passed the reference-cPPG validity criteria, whereas Table 2 reports candidate-extraction coverage over all 1378 evaluation cycles. The main detector returned an *e* candidate for all 1075 reconstructed cycles, but polarity-audit status was unavailable for one reconstructed candidate. Consequently, the positive-*e* percentage for the reconstructed signal was calculated using the 1074 candidates with available polarity-audit status.

Signal	Cycles	$T_{d}$ Local Peak (%)	$T_{d}$ Fallback (%)	Audit Status Unavailable (%)	Descending $T_{d}$, n (%)	*e* Candidate Returned (%)	Positive *e* Among Auditable Candidates (%)
Reference cPPG	1075	30.88	53.21	15.91	572 (53.21)	67.16	62.19
Reconstructed	1075	5.02	94.98	0.00	1021 (94.98)	100.00	69.18

**Table 7:** Descriptive agreement analysis restricted to 37 cycles from four participants in which both the reconstructed signal and reference cPPG contained a strict post-notch local maximum. All fallback assignments were excluded. Because the cycles were repeated observations from only four participants, the reported statistics describe this restricted subset only; inferential confidence intervals were not calculated.

Index	MAE	Bias	Descriptive 95% LoA	Pearson *r*	ICC(2,1)
ΔT (ms)	31.48	−29.15	−128.22 to 69.93	0.622	0.322
SI (m/s)	0.727	0.669	−0.724 to 2.061	0.769	0.593
RI	0.108	0.101	−0.090 to 0.292	0.023	0.002
AIx (%)	10.84	10.10	−9.04 to 29.23	0.023	0.002

**Table 8:** Temporal errors of fiducial points.

Fiducial Point	MAE (ms)
Systolic peak	18.42
Dicrotic Notch	35.12
Post-notch $T_{d}$ candidate	34.18

**Table 9:** Amplitude errors of fiducial points.

Fiducial Point	MAE
Systolic peak	0.0004
Dicrotic Notch	0.0729
Post-notch $T_{d}$ candidate	0.0857

**Table 10:** Effect of 30-point round-trip interpolation on reference cPPG-derived index estimation.

Index	Cycles	Detection Preserved (%)	MAE	95th Percentile Error
ΔT (ms)	1075	100.00	5.86	7.84
SI (m/s)	1075	100.00	0.081	0.253
RI	1075	100.00	0.007	0.011
sAI	717	99.31	0.266	0.674
AIx (%)	1075	100.00	0.741	1.101

**Table 11:** Individual fiducial point errors introduced by 30-point round-trip interpolation of reference cPPG cycles.

Fiducial	MAE (ms)	Median (ms)	95th Percentile (ms)
$T_{s}$	0.83	0.00	3.92
Notch	1.47	0.00	3.92
$T_{d}$	6.04	0.00	7.84

**Table 12:** Cycle-level waveform similarity between reconstructed signals and reference cPPG on the self-collected dataset.

Metric	Value
Cosine Similarity	0.990±0.039
MAE	0.060±0.036
RMSE	0.076±0.057

**Table 13:** Quantitative comparison of cPPG-derived vascular indices on the UBFC-rPPG dataset. The height-dependent SI was excluded because subject height information was unavailable.

Vascular Indices	Reference (Mean ± Std)	Reconstructed (Mean ± Std)	MAE (Mean ± Std)
RI	0.256±0.130	0.189±0.031	0.112±0.102
sAI	−0.648±0.275	−0.743±0.115	0.106±0.086
AIx (%)	−74.40±13.0	−81.1±3.1	11.2±10.2

**Table 14:** Quantitative comparison of cPPG-derived vascular indices on the UBFC-rPPG dataset using the fiducial-stability-based representative analysis window. The height-dependent SI was excluded because subject height information was unavailable.

Vascular Indices	Reference (Mean ± Std)	Reconstructed (Mean ± Std)	MAE (Mean ± Std)
RI	0.252±0.089	0.189±0.032	0.082±0.052
sAI	−1.181±0.144	−1.189±0.103	0.144±0.131
AIx (%)	−74.80±8.93	−81.08±3.16	8.19±5.23

**Table 15:** External cross-dataset evaluation in which the model trained on the self-collected dataset was directly applied to UBFC-rPPG without UBFC-specific retraining.

Evaluation	Subjects	Cycles	MAE	Pearson *r*
Waveform	42	4187	0.3189	0.150
ΔT (s)	42	4055	0.0278	0.832
RI	42	4055	0.4057	0.002
sAI	42	3937	0.5647	−0.032
AIx (%)	42	4055	40.565	0.002

Note: The candidate-extraction rate under the adopted systolic–notch–diastolic detector was 54.84% for raw rPPG, 100.00% for the reconstructed signals, and 96.85% for the reference cPPG. Successful candidate extraction indicates that the detector returned ordered candidates satisfying the index-computation criteria and includes fallback post-notch assignments when no strict local maximum was identified. It therefore does not establish recovery of a physiologically distinct diastolic peak in every cycle. The strict-versus-fallback detector audit on the self-collected dataset is reported in Table 6. Values correspond to the final rppg2cppg_dense_subject_split_nonmun cross-dataset CSV.

**Table 16:** Quantitative comparison of cPPG-derived vascular indices on the self-collected dataset.

Vascular Indices	Reference (Mean ± Std)	Reconstructed (Mean ± Std)	MAE (Mean ± Std)
SI (m/s)	5.811±1.199	5.878±0.805	0.411±0.606
RI	0.263±0.109	0.343±0.034	0.102±0.086
sAI	−1.334±0.589	−1.070±0.165	0.503±0.443
AIx (%)	−73.661±10.938	−65.691±3.378	10.167±8.593

**Table 17:** Quantitative comparison of cPPG-derived vascular indices using the fiducial-stability-based representative analysis window on the self-collected dataset.

Vascular Indices	Reference (Mean ± Std)	Reconstructed (Mean ± Std)	MAE (Mean ± Std)
SI (m/s)	5.796±0.758	5.693±0.561	0.149±0.217
RI	0.269±0.064	0.344±0.024	0.082±0.053
sAI	−1.053±0.777	−1.075±0.050	0.539±0.601
AIx (%)	−73.076±6.401	−65.590±2.443	8.249±5.271

**Table 18:** Agreement analysis for the height-independent, detector-defined systolic-to-post-notch delay ΔT on the self-collected dataset.

Analysis Level	MAE	MAE 95% CI	*r*	*r* 95% CI	ICC	ICC 95% CI	Bias	Bias 95% CI	95% LoA
Subject-level median	5.39	1.96–9.80	0.995	0.970–0.999	0.977	0.930–0.994	2.45	−1.96–8.33	−13.43 to 18.33
Cycle-level	25.72	–	0.695	–	0.532	–	−12.74	–	−121.42 to 95.94

**Table 19:** Subject-level agreement analysis of cPPG-derived vascular indices on the self-collected dataset. All statistics were recomputed from the same subject-level dataframe using subject-median values; limits of agreement were calculated as bias ± 1.96 SD of the paired differences.

Metric	Subjects	Cycles	MAE [95% CI]	Pearson *r* [95% CI]	ICC(2,1) [95% CI]	Bias [95% CI]	SD Diff.	Lower LoA	Upper LoA
ΔT (ms)	8	1075	5.39 [1.96, 9.80]	0.995 [0.970, 0.999]	0.977 [0.930, 0.994]	2.45 [−1.96, 8.33]	8.10	−13.43	18.33
SI (m/s)	8	1075	0.135 [0.035, 0.286]	0.997 [0.983, 0.999]	0.959 [0.919, 0.989]	−0.088 [−0.260, 0.031]	0.229	−0.538	0.361
RI	8	1075	0.071 [0.045, 0.097]	0.569 [−0.226, 0.909]	0.176 [0.001, 0.404]	0.071 [0.045, 0.097]	0.041	−0.009	0.150
sAI	7	721	0.362 [0.274, 0.442]	−0.495 [−0.909, 0.412]	−0.114 [−0.508, 0.065]	0.263 [0.038, 0.431]	0.295	−0.315	0.842
AIx (%)	8	1075	7.06 [4.51, 9.73]	0.569 [−0.226, 0.909]	0.176 [0.000, 0.395]	7.06 [4.51, 9.73]	4.05	−0.88	15.00

**Table 20:** Cycle-level agreement between vascular indices derived from the reconstructed signals and the corresponding reference cPPG.

Vascular Indices	Pearson *r*	ICC(2,1)	Bias	95% LoA
SI (m/s)	0.805	0.744	0.067	−1.361 to 1.496
RI	0.235	0.089	0.080	−0.129 to 0.289
sAI	−0.026	−0.012	0.263	−0.944 to 1.471
AIx (%)	0.235	0.089	7.970	−12.932 to 28.872

**Table 21:** Cycle-level agreement of SDPPG amplitude ratios related to sAI on the self-collected dataset.

Component	Cycles	Reference Median [IQR]	Reconstructed Median [IQR]	MAE	Bias	Pearson *r*
b/a	721	−1.337 [−1.464, −1.242]	−1.031 [−1.132, −0.964]	0.392	0.203	−0.064
e/a	721	0.082 [−0.066, 0.213]	0.008 [−0.004, 0.016]	0.202	−0.060	0.011
sAI	721	−1.419 [−1.552, −1.268]	−1.051 [−1.151, −0.959]	0.503	0.263	−0.026

**Table 22:** Effect of additive white Gaussian noise applied to the input rPPG. Values are means over ten independent noise realizations. The reconstruction model was not retrained and the reference cPPG was not modified.

Input SNR	Detection (%)	Waveform *r*	Waveform MAE	Systolic (ms)	Notch (ms)	Diastolic (ms)
Clean	100.00	0.945	0.069	18.05	35.38	35.31
20 dB	100.00	0.945	0.070	17.92	36.65	36.17
15 dB	100.00	0.944	0.071	17.92	39.62	38.83
10 dB	100.00	0.940	0.075	18.11	48.09	46.07
5 dB	100.00	0.932	0.081	18.65	59.62	49.93
0 dB	100.00	0.918	0.089	19.39	68.68	50.89

**Table 23:** Subject-level vascular-index error under input rPPG noise injection. Values are means over ten independent noise realizations.

Input SNR	ΔT MAE (ms)	SI MAE (m/s)	RI MAE	sAI MAE	AIx MAE (%)
Clean	5.39	0.135	0.071	0.362	7.06
20 dB	5.54	0.137	0.079	0.354	7.94
15 dB	4.83	0.115	0.087	0.324	8.69
10 dB	6.79	0.140	0.106	0.302	10.61
5 dB	16.91	0.358	0.142	0.304	14.15
0 dB	25.91	0.533	0.179	0.321	17.85

**Table 24:** Spectral composition of the evaluation cycles (upper block, mean fraction of total cycle energy) and sensitivity of the pipeline to a narrowed upper cutoff of the input rPPG (lower block). Cutoffs above the 4 Hz baseline were not evaluable because the corresponding spectral content is absent from the retained rPPG signals.

**Band (Hz)**	**0.7–2**	**2–2.5**	**2.5–3**	**3–4**	**4–6**	**6–8**
Input rPPG (%)	43.66	0.52	2.65	7.10	0.19	0.001
Reference cPPG (%)	62.32	0.22	2.65	11.35	7.73	0.51
**Upper Cutoff**	**Input Detect. (%)**	**Waveform** ***r***	**Waveform MAE**	**Notch (ms)**	**RI Cycle *r***	**RI Cycle ICC**
Baseline (4 Hz)	91.44	0.9454	0.0685	35.38	0.235	0.089
3.0 Hz	94.14	0.9457	0.0693	35.99	0.230	0.065
2.5 Hz	94.51	0.9451	0.0705	36.41	0.215	0.047
2.0 Hz	95.07	0.9434	0.0721	37.20	0.164	0.029
1.5 Hz	95.72	0.9423	0.0729	37.25	0.140	0.024

**Table 25:** Subject-level agreement under leave-one-subject-out and subject-wise five-fold cross-validation. Parenthetical values in the LOSO column are 95% confidence intervals obtained from 5000 participant-level bootstrap resamples; participant IDs were resampled and each statistic, including Pearson *r*, was recalculated within every bootstrap replicate. Five-fold results are reported as point estimates.

Index	Statistic	LOSO (28 Subjects)	5-Fold (28 Subjects)
ΔT (ms)	MAE	15.55 (5.46–29.27)	15.62
	Pearson *r*	0.909 (0.811–0.992)	0.908
	ICC(2,1)	0.837 (0.683–0.982)	0.837
SI (m/s)	MAE	0.213 (0.106–0.345)	0.214
	Pearson *r*	0.963 (0.920–0.992)	0.962
	ICC(2,1)	0.926 (0.857–0.974)	0.926
RI	MAE	0.066 (0.048–0.086)	0.069
	Pearson *r*	0.354 (−0.028–0.687)	0.190
	ICC(2,1)	0.139 (−0.011–0.250)	0.083
sAI	MAE	0.345 (0.273–0.416)	0.337
	Pearson *r*	0.198 (−0.184–0.538)	−0.140
	ICC(2,1)	0.060 (−0.058–0.172)	−0.039
AIx (%)	MAE	6.62 (4.76–8.61)	6.92
	Pearson *r*	0.354 (−0.028–0.687)	0.190
	ICC(2,1)	0.139 (−0.011–0.250)	0.083
Cycles/subjects analyzed		4555/28	4555/28
Reconstructed candidate-extraction rate (%)		100.00	100.00

## Data Availability

The data presented in this study are not publicly available due to privacy and ethical restrictions but are available from the corresponding authors upon reasonable request.
